# Overlapping *SETBP1* gain-of-function mutations in Schinzel-Giedion syndrome and hematologic malignancies

**DOI:** 10.1371/journal.pgen.1006683

**Published:** 2017-03-27

**Authors:** Rocio Acuna-Hidalgo, Pelagia Deriziotis, Marloes Steehouwer, Christian Gilissen, Sarah A. Graham, Sipko van Dam, Julie Hoover-Fong, Aida B. Telegrafi, Anne Destree, Robert Smigiel, Lindsday A. Lambie, Hülya Kayserili, Umut Altunoglu, Elisabetta Lapi, Maria Luisa Uzielli, Mariana Aracena, Banu G. Nur, Ercan Mihci, Lilia M. A. Moreira, Viviane Borges Ferreira, Dafne D. G. Horovitz, Katia M. da Rocha, Aleksandra Jezela-Stanek, Alice S. Brooks, Heiko Reutter, Julie S. Cohen, Ali Fatemi, Martin Smitka, Theresa A. Grebe, Nataliya Di Donato, Charu Deshpande, Anthony Vandersteen, Charles Marques Lourenço, Andreas Dufke, Eva Rossier, Gwenaelle Andre, Alessandra Baumer, Careni Spencer, Julie McGaughran, Lude Franke, Joris A. Veltman, Bert B. A. De Vries, Albert Schinzel, Simon E. Fisher, Alexander Hoischen, Bregje W. van Bon

**Affiliations:** 1 Department of Human Genetics, Radboud Institute of Molecular Life Sciences, Radboud University Medical Center, Nijmegen, The Netherlands; 2 Language and Genetics Department, Max Planck Institute for Psycholinguistics, Nijmegen, The Netherlands; 3 Department of Human Genetics, Donders Centre for Neuroscience, Radboud University Medical Center, Nijmegen, The Netherlands; 4 University of Groningen, University Medical Center Groningen, Department of Genetics, Groningen, the Netherlands; 5 McKusick-Nathans Institute of Genetic Medicine, Johns Hopkins University, Baltimore, Maryland, United States of America; 6 GeneDx, Gaithersburg, Maryland, United States of America; 7 Institute of Pathology and Genetics (IPG), Gosselies, Belgium; 8 Department of Pediatrics and Rare Disorders, Medical University, Wroclaw, Poland; 9 Division of Human Genetics, National Health Laboratory Service and School of Pathology, Faculty of Health Sciences, University of the Witwatersrand, Johannesburg, South Africa; 10 Medical Genetics Department, Koç University School of Medicine (KUSOM), İstanbul, Turkey; 11 Medical Genetics Department, İstanbul Medical Faculty, İstanbul University, İstanbul, Turkey; 12 Medical Genetics Unit, Anna Meyer Children's University Hospital, Florence, Italy; 13 University of Florence, Genetic Science, Firenze, Italy; 14 División de Pediatría, Pontificia Universidad Católica de Chile and Unidad de Genética, Hospital Dr. Luis Calvo Mackenna, Santiago Chile; 15 Department of Pediatric Genetics, Akdeniz University Medical School, Antalya, Turkey; 16 Laboratory of Human Genetics, Biology Institute, Federal University of Bahia (UFBA), Bahia, Brazil; 17 Hospital Santa Izabel, Salvador-Bahia, Brazil; 18 CERES-Genetica Reference Center and Studies in Medical Genetics and Instituto Fernandes Figueira / Fiocruz, Rio de Janeiro, Brazil; 19 Center for Human Genome Studies, Institute of Biosciences, USP, Sao Paulo, Brazil; 20 Department of Medical Genetics, Children’s Memorial Health Institute, Warsaw, Poland; 21 Department of Clinical Genetics, Sophia Children's Hospital, Erasmus MC, Rotterdam, The Netherlands; 22 Institute of Human Genetics, University of Bonn, Bonn, Germany and Department of Neonatology and Pediatric Intensive Care, Children's Hospital, University of Bonn, Bonn, Germany; 23 Division of Neurogenetics, Kennedy Krieger Institute, Departments of Neurology and Pediatrics, The Johns Hopkins Hospital, Baltimore, Maryland, United States of America; 24 Abteilung Neuropädiatrie, Medizinische Fakultät Carl Gustav Carus, Technische Universität Dresden, Germany; 25 Division of Genetics & Metabolism, Phoenix Children’s Hospital, Phoenix, Arizona, United States of America; 26 Institute for Clinical Genetics, TU Dresden, Dresden, Germany; 27 Department of Genetics, Guy's and St. Thomas' NHS Foundation Trust, London, United Kingdom; 28 North West Thames Regional Genetics Unit, Kennedy Galton Centre, North West London Hospitals NHS Trust, Northwick Park & St Marks Hospital, Harrow, Middlesex, United Kingdom; 29 Neurogenetics Unit, Department of Medical Genetics School of Medicine of Ribeirao Preto, University of Sao Paulo, Sao Paulo, Brazil; 30 Institute of Medical Genetics and Applied Genomics, University of Tübingen, Tübingen, Germany; 31 Unité de foetopathologie, Hôpital Pellegrin, Place Amélie Raba Léon, Bordeaux, France; 32 Institute of Medical Genetics, University of Zurich, Schlieren, Switzerland; 33 Genetic Health Queensland, Royal Brisbane and Women's Hospital, Brisbane, Queensland and School of Medicine, The University of Queensland, Brisbane, Queensland, Australia; 34 Institute of Genetic Medicine, International Centre for Life, Newcastle University, Newcastle upon Tyne, United Kingdom; 35 Donders Institute for Brain, Cognition and Behaviour, Radboud University, Nijmegen, The Netherlands; 36 Department of Internal Medicine and Radboud Center for Infectious Diseases (RCI), Radboud University Medical Center, Nijmegen, The Netherlands; Stanford University School of Medicine, UNITED STATES

## Abstract

Schinzel-Giedion syndrome (SGS) is a rare developmental disorder characterized by multiple malformations, severe neurological alterations and increased risk of malignancy. SGS is caused by *de novo* germline mutations clustering to a 12bp hotspot in exon 4 of *SETBP1*. Mutations in this hotspot disrupt a degron, a signal for the regulation of protein degradation, and lead to the accumulation of SETBP1 protein. Overlapping *SETBP1* hotspot mutations have been observed recurrently as somatic events in leukemia. We collected clinical information of 47 SGS patients (including 26 novel cases) with germline *SETBP1* mutations and of four individuals with a milder phenotype caused by *de novo* germline mutations adjacent to the *SETBP1* hotspot. Different mutations within and around the *SETBP1* hotspot have varying effects on SETBP1 stability and protein levels *in vitro* and in *in silico* modeling. Substitutions in SETBP1 residue I871 result in a weak increase in protein levels and mutations affecting this residue are significantly more frequent in SGS than in leukemia. On the other hand, substitutions in residue D868 lead to the largest increase in protein levels. Individuals with germline mutations affecting D868 have enhanced cell proliferation *in vitro* and higher incidence of cancer compared to patients with other germline *SETBP1* mutations. Our findings substantiate that, despite their overlap, somatic *SETBP1* mutations driving malignancy are more disruptive to the degron than germline *SETBP1* mutations causing SGS. Additionally, this suggests that the functional threshold for the development of cancer driven by the disruption of the SETBP1 degron is higher than for the alteration in prenatal development in SGS. Drawing on previous studies of somatic *SETBP1* mutations in leukemia, our results reveal a genotype-phenotype correlation in germline *SETBP1* mutations spanning a molecular, cellular and clinical phenotype.

## Author summary

Several Mendelian disorders are caused by germline *de novo* mutations in genes in which somatic mutations have been implicated as cancer driver mutations. Germline *de novo* mutations in a hotspot of *SETBP1* cause Schinzel-Giedion syndrome (SGS), a rare developmental disorder characterized by neurological alterations, malformations and increased cancer risk. Overlapping somatic *SETBP1* mutations have been identified recurrently in myeloid leukemia. In this study, we characterize at the molecular and clinical level the largest cohort yet of individuals with SGS. We analyze the distribution and magnitude of effect of germline and somatic *SETBP1* mutations in SGS and leukemia, respectively. *SETBP1* mutations with weak effect are almost exclusively germline events, while strongly activating *SETBP1* mutations occur both in SGS and leukemia. Strikingly, most cancer cases in SGS are observed in patients with strongly activating germline *SETBP1* mutations. Our findings support a genotype-phenotype correlation for SGS and suggest the existence of a functional threshold required to drive malignancy both for germline and somatic *SETBP1* mutations. This finding could be extrapolated to mutations in other genes implicated in developmental disorders and cancer, showing that the fields of cancer and developmental genetics can learn from the other discipline to gain insight into their own subject.

## Introduction

Schinzel-Giedion syndrome (SGS; OMIM 269150) is a rare developmental disorder characterized by multiple malformations including midface hypoplasia, cardiac defects, hydronephrosis and skeletal abnormalities [1–3]. This clinically recognizable syndrome was the first dominant disorder for which the underlying genetic cause was identified by whole exome sequencing [4]. In 12 of 13 unrelated individuals with this disorder, we identified germline *de novo* mutations in *SETBP1* clustering to a hotspot of 12 base pairs coding for residues 868 to 871 of the SETBP1 protein [4]. Interestingly, shortly after the identification of germline *de novo* mutations in *SETBP1* as the cause of SGS, overlapping somatic mutations in *SETBP1* were reported in several types of myeloid malignancies [5–7]. This dual role in cancer and development is not unique to *SETBP1*; a growing number of genes in which germline mutations cause developmental disorders, such as *HRAS*, *ASXL1*, *EZH2* and *FGFR2*, are also known to harbor overlapping somatic mutations which drive the development of cancer [8]. This genetic overlap is not entirely unexpected; higher rates of childhood cancer have been identified in individuals with birth defects and *vice versa* [9–11], a finding which is thought to be the consequence of abnormalities in molecular pathways shared between embryogenesis and cancer development [12,13].

The precise function of *SETBP1*, which encodes the SET-binding protein 1 (OMIM 611060), is yet to be discovered and, as a result, the molecular consequences of *SETBP1* mutations remain largely unknown. However, the clustering of all germline *SETBP1* mutations identified in SGS to a single region and their overlap with the somatic events identified in myeloid malignancies support a gain-of-function effect on the SETBP1 protein. This recurrently mutated region of the protein is highly conserved and has been identified as a degron signal targeted by the SCF-βTrCP1 E3 ligase [5]. A degron is a peptide sequence that is recognized and bound by a component of the ubiquitin-proteasome pathway, thereby initiating degradation of the protein by ubiquitination [14]. As a result, mutations localizing to the degron in SETBP1 disrupt binding by the βTrCP1 E3 ligase, increase protein stability by interfering with ubiquitination [15] and ultimately lead to accumulation of SETBP1 protein in cells [5]. While the molecular consequences of germline *SETBP1* mutations are poorly understood, somatic mutations disrupting the SETBP1 degron lead to increased proliferation in myeloid progenitors [7], possibly mediated by effects on its interaction partner SET, phosphorylation of PP2A and transcriptional activation of *HOXA9* and *HOXA10* [5,16,17].

Additional clinical and functional investigation is warranted to gain more understanding about the molecular mechanisms of SGS. Here we present the clinical characterization of the largest cohort of individuals with genetically confirmed SGS and establish genotype-phenotype correlations for SGS. Given the occurrence of overlapping germline and somatic *SETBP1* mutations, we compare the mutations in SGS and leukemia to identify genetic and functional differences between *SETBP1* mutations in both conditions.

## Results

### Clinical features of SGS

**Fig 1:**
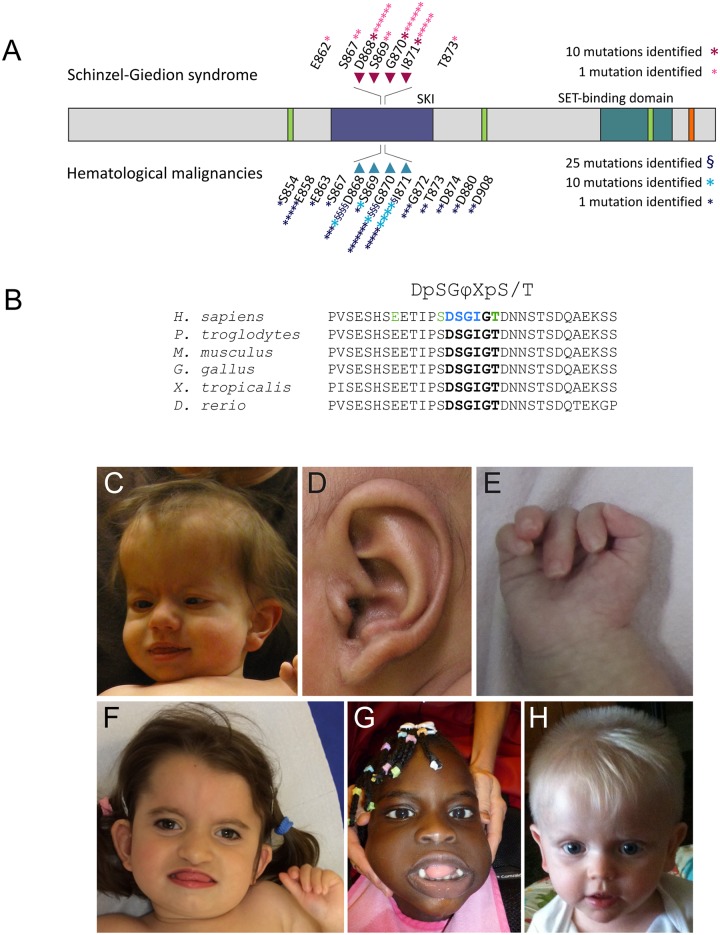
Genetic and clinical characteristics of individuals with germline *SETBP1* mutations and Schinzel-Giedion syndrome. **A**. Schematic representation of the SETBP1 protein, indicating changes found in SGS and in hematologic malignancies. The residues of the canonical degron are highlighted with arrows. Protein domains of SETBP1 are shown in different colors with green corresponding to three AT hooks, purple to the SKI homologous region, blue to the SET binding domain and orange to a repeat domain (modified from Piazza *et al*.). **B**. Sequence alignment of the region containing the degron of SETBP1 (in bold) in human (Uniprot accession number Q9Y6X0), chimpanzee (H2QEG8), mouse (Q9Z180), chicken (A0A1D5PT15), african clawed frog (F6TBV9) and zebrafish (B0R147). The consensus motif for βTrCP1 substrates is shown on top, with φ representing a hydrophobic residue and X any amino acid. Residues in which pathogenic germline mutations have been identified in classic SGS are highlighted in blue, while residues in which novel mutations leading to an atypical form of SGS are shown in green. **C**. Distinctive facial features encountered in classic SGS (current case 9 at 1,5 years of age). **D**. Typical question mark-shaped ear observed in current case 18. **E**. Characteristic hand posture with clenched fingers from current case 16. **F**. Facial features of current case 27 with a mutation in SETBP1 residue S867 at 4 years of age. Note the clenched fingers. **G**. Facial features of current case 28 with a mutation in SETBP1 residue E862 at 5 years of age. **H**. Facial features of current case 29 with a mutation in SETBP1 residue T873 at the age of 23 months.

Classic SGS is caused by mutations within four residues of the SETBP1 degron (D868, S869, G870 and I871, Fig 1A and 1B), constituting the critical consensus sequence of the degradation signal [18] (from here on, referred to as the canonical degron). Since the initial description of 12 mutation-positive cases [4], we have gathered clinical details of 26 additional individuals with SGS genetically confirmed by the presence of *de novo* mutations in *SETBP1* (see Table 1 and S1 Table). In addition, we present three patients with a milder phenotype variably overlapping with SGS and secondary to novel mutations in *SETBP1* affecting highly conserved residues in close proximity to the canonical degron (current cases 27–29, with mutations in residues E862, S867 and T873 shown in green in Fig 1B). We report on the clinical features observed in our cohort in addition to previously published mutation-positive cases to further delineate this disorder (n = 51 individuals).

**Table 1:** Major clinical findings in 51 individuals with germline mutations in *SETBP1*. NA stands for “Not Assessed”.

Residue affected in SETBP1	E862	S867	D868	S869	G870	I871	T873	All degron-affecting mutations (868–871)	
Male(M):female(F)	1F	2 F	8F:7 M	2F	5F: 10M	6F:9M	1M	21F:26M	
**Craniofacial findings**									
Microcephaly	1/1	1/2	10/12	1/2	10/13	8/11	0/1	29/39	74.4%
SGS facial gestalt	0/1	2/2	15/15	2/2	15/15	15/15	0/1	47/47	100.0%
**Congenital anomalies**									
Hydronephrosis	0/1	0/2	15/15	2/2	14/15	14/15	0/1	45/47	95.7%
Genital abnormalities	1/1	0/2	14/15	1/2	14/15	12/13	0/1	41/45	91.1%
Cardiac defects	0/1	1/2	10/15	1/2	4/13	5/13	0/1	20/43	46.5%
Tracheo/laryngomalacia	0/1	0/1	3/4	0/2	3/8	2/2	0/1	8/16	50.0%
Inguinal hernia	0/1	0/1	2/4	0/2	6/8	0/1	0/1	8/15	53.3%
Alacrima	0/1	2/2	6/10	0/2	7/9	6/6	0/1	19/27	70.4%
**Neurodevelopmental anomalies**									
Developmental delay	1/1	2/2	14/14	2/2	13/13	10/10	1/1	39/39	100.0%
Seizures	0/1	2/2	15/15	2/2	13/14	12/13	0/1	42/44	95.5%
Spasticity and/or hypertonia	0/1	1/1	4/4	1/2	8/10	4/4	1/1	17/20	85.0%
Vision impairment	1/1	1/1	7/10	1/2	7/8	5/6	0/1	20/26	76.9%
Hearing impairment	0/1	0/1	9/9	0/1	7/8	8/9	0/1	24/27	88.9%
Progressive failure to thrive	0/1	0/1	10/11	1/2	13/13	8/9	0/1	32/35	91.4%
**Brain MRI/CT**									
Ventriculomegaly	0/1	NA	6/12	2/2	11/14	7/14	0/1	26/42	61.9%
Underdeveloped corpus callosum	0/1	NA	9/11	0/2	12/13	10/12	0/1	31/38	81.6%
Cortical atrophy or dysplasia	0/1	NA	8/10	0/2	7/10	3/11	1/1	18/33	54.5%
Choroid plexus cysts	0/1	NA	2/9	0/2	8/10	3/10	0/1	13/31	41.9%
**Radiological findings**									
Sclerotic base of skull or mastoid	NA	NA	9/10	0/1	5/5	5/7	NA	19/23	82.6%
Hypoplastic distal phalanges	0/1	NA	8/9	0/1	8/9	5/6	NA	21/25	84.0%
Broad ribs	0/1	NA	10/13	2/2	6/7	9/9	NA	27/31	87.1%
Hypoplastic/underossified pubic bones	0/1	NA	6/7	2/2	4/5	6/6	NA	18/20	90.0%
**Tumors**	0/1	0/2	5/11	0/2	1/11	1/9	0/1	7/33	21.2%

#### Dysmorphic facial features

All individuals with SGS have characteristic facial features that are easily recognizable (Fig 1C and S1 Fig). Typically, SGS patients have large fontanelles (n = 37/41), a prominent forehead (n = 41/44), bitemporal narrowing (n = 32/36), shallow orbits or prominent eyes (n = 36/38), hypertelorism (n = 36/42) a retracted and shortened midface (n = 45/45) and full cheeks, leading to a facial frontal silhouette in the shape of a number eight [19]. Additionally, most patients have a deep groove under the eyes (n = 38/39), upslanting palpebral fissures (n = 21/26) and a short nose with a bulbous nasal tip (n = 43/44). Some individuals may present with less recognizable facial features in the first weeks after birth and after the age of 18 months. In those cases, additional diagnostic clues that may facilitate the diagnosis include the abnormal shape of the ears (n = 38/39). Classically, the ears of individuals with SGS are low-set and posteriorly rotated with anteriorly angulated lobules giving rise to a question mark shape (Fig 1D). In individuals who do not have the typical lobules, the majority does have folded helices and prominent anti-helices. About half of the patients show a large mouth (n = 17/33) with an everted lower lip and a protruding tongue (n = 18/38). In addition, they may present with micrognathia (n = 29/30) and a philtrum groove. Hypertrichosis was identified in two thirds of the patients (n = 26/37), facial hemangioma in eight of 33 cases and a short neck in 30 of 33 cases.

#### Skeletal features

As molecular testing for *SETBP1* mutations has become available, the extent of diagnostic radiologic evaluations has diminished and, therefore, has not been performed in all patients within this cohort (data were available for 31 cases). Skeletal characteristics present in over 75% of these patients included a sclerotic base of the skull (n = 19/23) with wide occipital synchondrosis (n = 18/23), widening of ribs (n = 27/31), short pubic rami, wide pubic symphysis (n = 18/20) and hypoplastic distal phalanges in hands and feet (n = 21/25). In case 22, the synchondrosis had closed completely in a later radiologic examination. Post-axial polydactyly was noted in only 11% of patients (n = 4/38). Retrospective study of photographs of the hands of individuals with SGS shows that a typical posture with clenched fingers is common (Fig 1E and 1F).

#### Neurologic features

Microcephaly was observed in approximately two thirds of the individuals with SGS (n = 29/39). The occipitofrontal circumference in the remaining individuals for whom data were available was always below the 50^th^ percentile and often near the 10^th^ percentile. Severe developmental delay occurs in all individuals with SGS and 95% present with epilepsy (n = 42/44). Almost all common types of epilepsy occur and seizures are characteristically extremely refractory to treatment with medication or ketogenic diet. Many patients have hearing (n = 24/27) or vision impairment (n = 20/26) thought to be of cerebral origin. Spasticity was noted in 17 out of 20 of cases.

Structural brain abnormalities are variable in SGS. The most common anomaly is hypoplasia or aplasia of the corpus callosum (n = 31/38). Additional abnormalities often encountered are cortical atrophy (n = 18/33), ventricle anomalies (n = 26/42), abnormal gyration and delayed myelination and choroid plexus cysts (n = 13/31).

#### Additional congenital anomalies

Individuals with SGS nearly always present hydronephrosis (n = 45/47), a feature that can be detected during routine prenatal medical exams. Two patients in our cohort were noted to have hydronephrosis at 20 weeks of gestation. Other kidney anomalies include abnormal ureters, renal cysts and stones. Almost all patients have genital anomalies (n = 41/45), which include hypospadias, underdeveloped genitalia and displaced anus. Half of the patients (n = 20/43) have structural cardiac malformations, the majority of which present with defects of the atrial septum. Other anomalies include patent foramen ovale, patent ductus arteriosus and cardiac hypertrophy. Alterations in the internal organs may be identified in some individuals with SGS, including hypoplasia of the pancreatic tail or hepatosplenomegaly. Microscopic evaluation in one patient (current patient 6) showed dilated glands and mucus depositions in intra-acinar pancreatic ducts at a post-mortem examination at 4 days of age. The observed features were similar to the mucus obstruction seen in cystic fibrosis. However, *CFTR* analysis in this patient proved negative. Nineteen patients (n = 19/27) were noted to have alacrima. Inguinal hernia (n = 8/15) and talipe(s) equinovarus (n = 11/17) were also frequently noted.

#### Swallowing and breathing difficulties

A major medical problem encountered in the care of individuals with SGS is the difficulty in swallowing and breathing. This is caused by a combination of factors such as structural abnormalities of the respiratory apparatus (*e*.*g*. choanal stenosis, n = 10/34), tracheobronchomalacia (n = 8/16), lung hypoplasia, poor management of oral and respiratory secretions (*e*.*g*. resulting from micrognathia, gingiva hypertrophy or excessive mucus production) and a high susceptibility to airway infections. Although gingiva hypertrophy can result from the use of anti-epileptic medication, this feature was already noted in a newborn with SGS at four days of age (patient 6). In this case, dilated laryngeal and broncheal glands filled with mucus were observed in the microscope, consistent with a previous report of thickened alveolar mucosa and fibrous hyperplasia of the gingiva with mucoid depositions [20], suggesting that this is a feature of SGS.

#### Cause of death

Most affected individuals do not survive past childhood, with pneumonia as a major cause of death in SGS (n = 8/15). Other reported causes in early infancy include congenital cardiac defects, tumors, lung hypoplasia, intractable seizures and sudden cardiac arrest. Six individuals developed solid tumors, predominantly of neuroepithelial origin in the lumbosacral region. Additionally, one individual in this cohort developed juvenile myelomonocytic leukemia. Although the majority of individuals die during infancy, five out of twelve patients with a protein substitution in residue G870 lived to the age of 5 or older (5 to 15 years of age). The average age of death of deceased individuals with a substitution in D868, S869, G870 and I871 is 18 months (n = 10), 32 months (n = 2), 48 months (n = 7) and 25 months (n = 8), respectively (see S2 Fig). Due to the small size of the cohort, it is not possible to draw conclusions on whether there are differences in survival depending on the mutated residue.

#### Individuals with mutations outside of the degron

Individuals with germline *SETBP1* mutations occurring outside the degron (n = 4) are reported in this separate section. Both patients with a mutation in residue S867 (one reported in [21] and current case 27 from our cohort, see Fig 1F and S3A–S3D Fig) had a characteristic facial appearance, genital anomalies and seizures but did not show hydronephrosis. Other features of these patients fit within the spectrum of SGS and are summarized in Table 1 and S1 Table.

One individual with a mutation in residue E862 did not have a characteristic facial appearance (Fig 1G and S3E–S3H Fig), seizures nor hydronephrosis. However, this patient had several other overlapping features with SGS, including dysphagia requiring a gastrostomy tube, vision loss due to retinal dystrophy, bilateral renal cysts and severe spasticity. She showed microcephaly, prognathism, small feet with short toes and a normal height. At five years of age, she had severe intellectual disability and could neither speak nor walk.

The individual with a mutation in residue T873 had the mildest phenotype, presenting with developmental delay, autistic features, spastic diplegia and milder dysmorphic features (Fig 1H and S3I–S3L Fig). This patient does not present any of the major congenital anomalies commonly found in SGS and, despite developmental delay, his initial developmental outcome seemed higher compared to individuals with *SETBP1* mutations within the degron. He achieved several milestones: smiling at eight weeks, making vowel sounds at 14 months, sitting unassisted at 22 months and, at 2 years of age, he was able to maintain crawling position, walk with help and feed himself. Thereafter, he entered a regression phase with loss of the aforementioned milestones and started self-injurious behavior. An IQ test was not available but, at 4 years of age, his functional level was estimated to be that of an eight-month-old.

### Functional characterization of SETBP1 variants

Mutations within the canonical degron of SETBP1 disrupt its interaction with βTrCP1, increasing protein stability and SETBP1 levels [5,15] but the effect of *SETBP1* mutations outside the canonical degron on protein stability is unknown. Clear differences in the severity of the phenotype resulting from germline *SETBP1* mutations within and outside the canonical degron suggest a difference in the effect of the mutations depending on their localization. Therefore, we performed the functional characterization of the most frequent *SETBP1* mutations observed within the canonical degron and causative for classic SGS (D868N, S869N, G870S and I871T) as well as one mutation outside the canonical degron leading to atypical SGS (E862K).

#### Effects of SETBP1 variants on protein levels and protein stability

**Fig 2:**
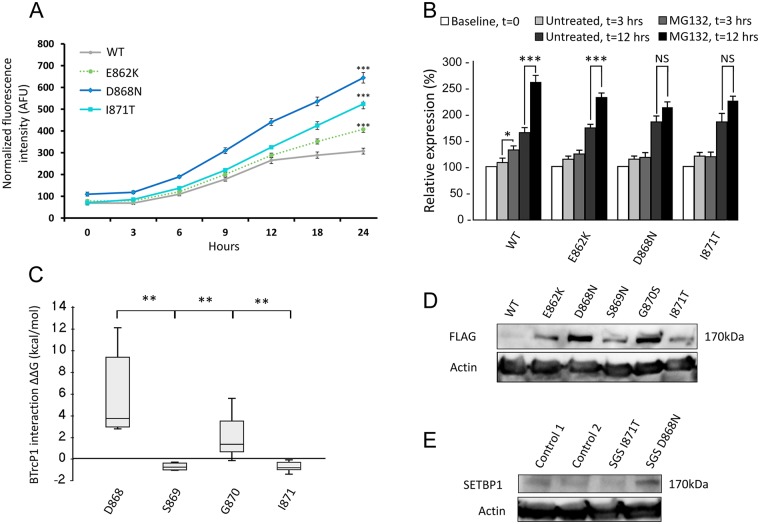
Functional analysis of SETBP1 mutations identified in SGS. **A**. Fluorescence measurements in live HEK293 cells expressing YFP-tagged SETBP1 variants. (*** p<0.001 versus wild-type and all mutants, ANOVA). All SETBP1 mutations studied displayed a statistically significant difference compared to wild-type and to all other mutations. This graph is representative of 3 independent experiments performed, with 6 technical replicates per experiment. Bars represent the standard error. **B**. Relative expression of SETBP1 protein variants in live HEK293 cells treated with MG132 proteasome inhibitor or vehicle only. Bars represent the standard error. (*** p<0.001, * p<0.05, NS: not significant, Student’s T test and Mann-Whitney U test). **C**. ΔΔG values for degron-βTrCP1 interaction for all germline mutations reported in SETBP1 per residue (** p<0.01 D868 versus other residues; ANOVA). **D**. Immunoblot of whole cell lysates of HEK293 cells expressing FLAG-tagged SETBP1 variants probed with anti-FLAG antibody. **E**. Immunoblot of whole-cell lysates of fibroblasts probed with anti-SETBP1 antibody. Fibroblasts were derived from two cases of SGS, one carrying the I871T variant and the other carrying the D868N variant, as well as from two unrelated controls. In D and E, blots were stripped and re-probed with anti-β-actin antibody.

To investigate the effect of SETBP1 variants on protein stability, we quantified expression levels of YFP-fusion proteins in live HEK293 cells based on fluorescence intensity (S4 Fig). All three pathogenic SETBP1 variants E862K, D868N and I871T showed increased protein levels compared to WT SETBP1 (Fig 2A; p<0.001, ANOVA). Interestingly, SETBP1 variants occurring within the canonical degron (D868N, I871T) showed higher protein levels compared to the variant adjacent to the degron sequence (E862K; p<0.001 versus other mutants, ANOVA).

To verify that the increased protein levels seen in cells expressing pathogenic SETBP1 variants are due to resistance to degradation by the proteasome, we treated transfected HEK293 cells with MG132, a proteasome inhibitor (Fig 2B). WT SETBP1 protein levels were sensitive to inhibition of the proteasome, as evidenced by a significant increase in fluorescence intensity after MG132 treatment for 3 hours (132% vs 108% for treated versus untreated cells compared to baseline, p<0.05, Student’s T-test). The difference in expression levels after 3 hours of MG132 treatment was not significant for SETBP1 variants E862K, D868N and I871T (E862K: 126% vs 113%; D868N: 118% vs 115%; I871T: 121% vs 121% for treated versus untreated cells compared to baseline). Prolonged treatment with MG132 resulted in a larger fold increase in fluorescence observed for treated versus untreated cells in cells transfected with WT versus mutant SETBP1 (1.58 for WT, 1.32 for E862K, 1.14 for D868N and 1.21 for I871T; p<0.01, ANOVA). Indeed, prolonged MG132 treatment results in a significant increase in expression levels of wildtype SETBP1 and the E862K mutant (SETBP1: 261% vs 164%; E862K: 230% vs 173% for treated versus untreated cells compared to baseline, p<0.001, Student’s T test). Prolonged MG132 treatment did not significantly affect the expression levels of the D868N and I871T SETBP1 variants (211% vs 185% and 225% vs 186% for treated versus untreated cells compared to baseline, respectively). Cycloheximide chase of wild-type and mutant SETBP1 suggests decreased degradation of mutant SETBP1 as compared to the wild-type protein (S5 Fig), further supporting that mutations in the SETBP1 degron confer increased stability to the protein. This suggests that pathogenic SETBP1 variant proteins have decreased sensitivity or resistance to proteasome inhibition with varying magnitude of effects.

#### Different magnitude of effect of SETBP1 variants within the canonical degron

To explore the effect of disease-causing SETBP1 variants on the interaction of SETBP1 with βTrCP1, we performed *in silico* modeling of all known germline mutations that occur within the canonical degron (D868-I871) identified in patients with classic SGS. Although the protein structure of SETBP1 is not known, the sequence of the βTrCP1 binding site in the SETBP1 degron is similar to the sequence of a degron in β-catenin (S2 Table) [18]. We therefore used a protein model of the degron of β-catenin in complex with βTrCP1 as a template to analyze the effect of pathogenic germline *SETBP1* variants on molecule stability and on the degron/βTrCP1 interaction energy (molecule ΔΔG and βTrCP1 interaction ΔΔG, respectively; Fig 2C and S3 Table). Modeling of all pathogenic germline substitutions observed in the degron shows that the interaction with βTrCP1 was most affected for substitutions of the aspartate residue from the motif (p<0.01 versus other residues, ANOVA), which is in line with previous findings [18]. This would entail that among the variants observed in SGS, mutations in residue D868 would have the largest effect on degron/βTrCP1 interaction, followed by mutations in G870.

To verify the differences on protein stability observed at the computational level, we used immunoblotting to examine the protein levels of SETBP1 variants in transfected HEK293 cells. Pathogenic SETBP1 variants led to substantially higher protein levels than WT SETBP1 (Fig 2D and S6 Fig). Variants D868N and G870S resulted in dramatically increased protein levels, whereas E862K, S869N and I871T showed a more modest increase in protein levels (Fig 2D). To further explore these findings, we performed immunoblotting to examine endogenous SETBP1 protein levels in fibroblasts derived from individuals with SGS. Fibroblasts from an individual carrying the D868N variant showed increased SETBP1 protein levels compared to two unrelated age-matched controls, whereas fibroblasts from a patient carrying the I871T mutation did not show any differences in endogenous SETBP1 levels compared to the controls (Fig 2E). Analysis of mRNA levels showed decreased *SETBP1* mRNA levels in cells of individuals with SGS as compared to controls, suggesting that the increase observed in endogenous SETBP1 protein does not result from increased *SETBP1* transcription (S7 Fig). Together, these results suggest that SETBP1 degron mutations have variable effects on protein stability, with the D868N variant having a stronger effect than I871T.

#### Overlapping SETBP1 mutations in SGS and in myeloid malignancies

**Fig 3:**
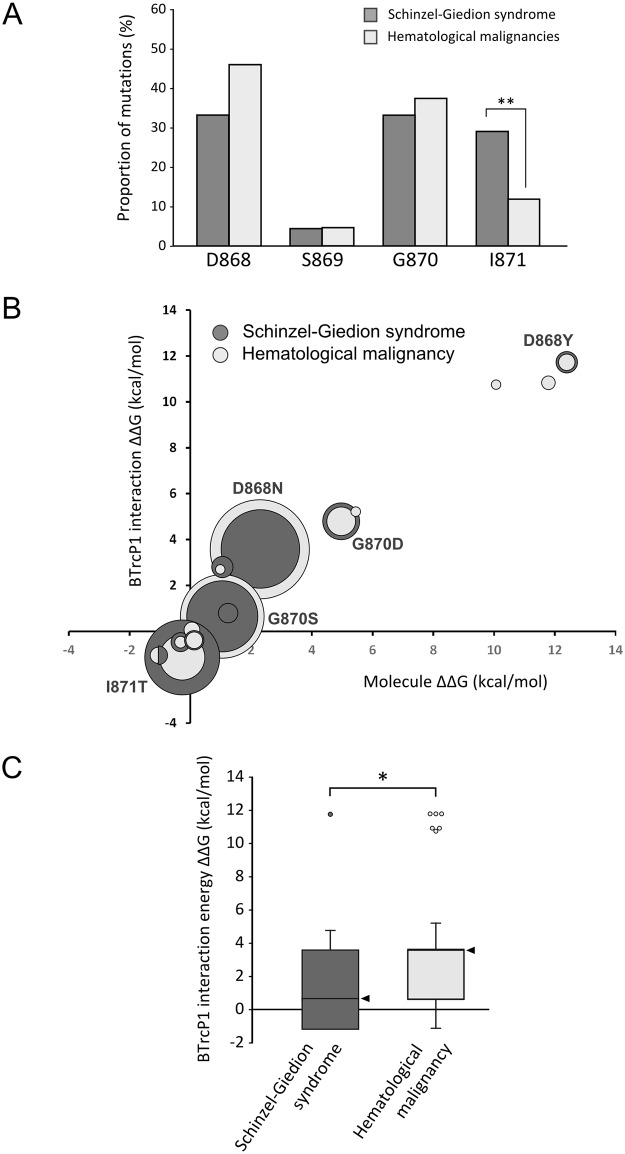
On average, *SETBP1* mutations seen in cancer are more severe than those observed in SGS. **A**. Distribution of mutations within the SETBP1 degron in SGS and in hematological malignancies. (** p<0.01, Fisher’s test and Bonferroni correction for multiple testing). **B**. ΔΔG values for protein stability (x-axis) and degron-βTrCP1 interaction (y-axis) for all mutations reported in SETBP1. The size of each circle is proportional to the frequency of the mutation in each condition. **C**. Difference in free energy of binding in the interaction between βTrCP1 and the degron of variants arising from germline or somatic SETBP1 mutations compared to that of the interaction between βTrCP1 and the wild-type degron (* p <0.05, Mann-Whitney’s U test). The median is highlighted by an arrow head.

Overlapping mutations in *SETBP1* have been identified as germline *de novo* events in SGS and as somatic mutations in myeloid malignancies (Fig 1A). Considering that canonical degron mutations vary in the magnitude of their effect, we compared germline and somatic *SETBP1* mutations to detect differences between the mutations in both conditions. In total, 48 germline *de novo* mutations within the SETBP1 degron have been reported in individuals with SGS, both from our cohort and from published literature (see S4 Table) [4,21–29]. Similarly, we have retrieved from literature 245 individual somatic mutations in the SETBP1 degron, associated with different myeloproliferative disorders (see S4 Table) [5–7,30–48]. While the mutations overlap in both conditions, the distribution of the mutations within the canonical degron sequence is not the same; a significantly higher number of mutations affect residue I871 in SGS cases compared to myeloid malignancy cases (29% and 12%, respectively, p<0.01, Fisher’s test with Bonferroni correction; see Fig 3A).

All SETBP1 degron mutations identified in leukemia were compared to mutations observed in SGS on their effect on the SETBP1-βTrCP1 interaction using *in silico* modeling (Fig 3B). Taking into account the frequency of each mutation, we then compared the difference in degron-βTrCP1 interaction energy for SETBP1 mutations observed in SGS versus those observed in myeloid malignancies (Fig 3C). Generally, mutations observed in myeloid malignancies showed higher ΔΔG values than mutations observed in SGS (p <0.05, Mann-Whitney’s U test). However, the difference in ΔΔG between germline and somatic *SETBP1* mutations after exclusion of mutations in codon I871 is no longer statistically significant. This suggests that the difference between ΔΔG values between germline and somatic *SETBP1* mutations may be secondary mainly to the prevalence of mutations at codon I871 in each condition.

#### Similarities in downstream consequences of germline and somatic SETBP1 mutations

**Fig 4:**
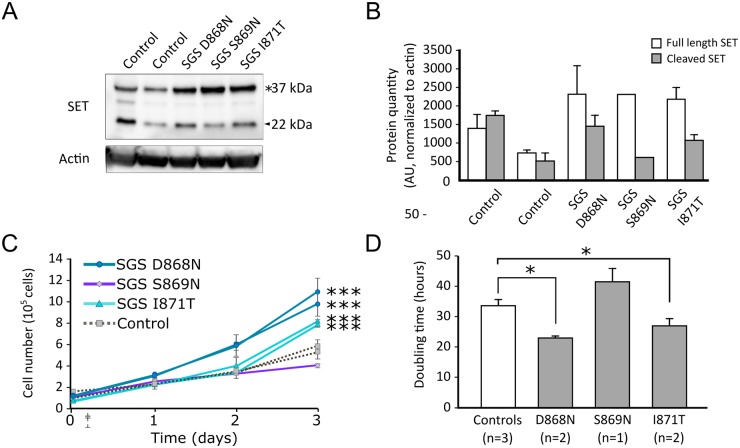
Increased SET protein and proliferation in cell lines of patients with SGS. **A**. Immunoblot of whole cell lysates of lymphoblastoid cell lines (LCLs) derived from three SGS patients and two age-matched controls. The blot was probed with anti-SET, before stripping and re-probing with β-actin to confirm equal loading. * denotes full-length SET protein (37kDa), arrowhead denotes cleaved SET protein (22kDa). **B**. SET protein levels as determined by densitometric analysis of Western blot data and normalized to actin (n = 2 independent experiments). Bars represent the standard error. **C**. Proliferation assay of LCLs derived from SGS individuals and age-matched controls over 3 days (*** p< 0.001 versus controls, ANOVA). Bars represent the standard error. **D**. Mean doubling time for LCLs derived from individuals with germline *SETBP1* mutations compared to controls over 3 days (* p<0.05 versus controls, ANOVA). The mean of two experiments is shown, bars represent the standard error.

The SETBP1-SET interaction regulates SET protein levels and can induce cleavage of SET [16]. Higher levels of SET protein have been reported with overexpression of wildtype SETBP1 in HEK cells and of SETBP1 G870S in TF1 cells [5,16]. To establish whether pathogenic germline *SETBP1* degron mutations D868N, S869N and I871T also lead to increased SET protein levels in SGS, we performed immunoblotting on protein lysates of lymphoblastoid cell lines (LCLs) derived from three unrelated patients with SGS. Compared to age-matched controls, we observed that cell lines derived from individuals with germline mutations in the *SETBP1* degron show higher SET protein levels, with an increase in full length versus cleaved SET protein (Fig 4A and 4B).

Somatic mutations in *SETBP1* drive the development of myeloid malignancies by increasing proliferation in leukemic cells [15]. We examined LCLs derived from individuals with germline *SETBP1* mutations seen in SGS to determine whether they presented increased proliferation. In a time course experiment, we observed that LCLs derived from individuals with SGS proliferate faster and have shorter doubling times than cells derived from age-matched controls in a genotype-dependent manner (p<0.001 versus controls by ANOVA, see Fig 4C and 4D). LCLs from unrelated individuals carrying D868N mutations had the shortest doubling times, followed by LCLs from unrelated patients carrying I871T mutations and by cells derived from age-matched controls (23.2, 27 and 33.8 hours respectively, p<0.05, ANOVA). LCLs from an individual with a S869N mutation consistently showed lower proliferation and longer doubling time (41.7 hours; Fig 4D). RNA sequencing of LCLs of individuals with germline *SETBP1* mutations revealed differential expression of 1811 genes between controls and individuals with SGS (S8 Fig), of which 632 were upregulated and 1179 were downregulated [49]. Gene set enrichment analysis of differentially expressed genes between controls and individuals with germline *SETBP1* mutations shows an enrichment for genes involved in mRNA transcription and translation, mitochondrial respiration and cell cycle (S5 Table) [50].

#### Increased tumorigenesis in individuals with mutations in residue D868

To determine whether a correlation exists between the magnitude of effect of a germline *SETBP1* mutation and tumorigenesis in individuals with SGS, we examined the clinical data of our cohort and previously published mutation-positive cases. In total, 7 malignancies have been reported in mutation-positive individuals, of which 5 occurred in patients with mutations in residue D868 (four tumors of primitive neuroectodermal origin and one myeloid leukemia). The remaining tumors included one ependymal tumor with myxopapillary and ependymoblastic differentiation in an individual with mutation G870D and one primitive neuroectodermal tumor in an individual with mutation I871T.

**Fig 5:**
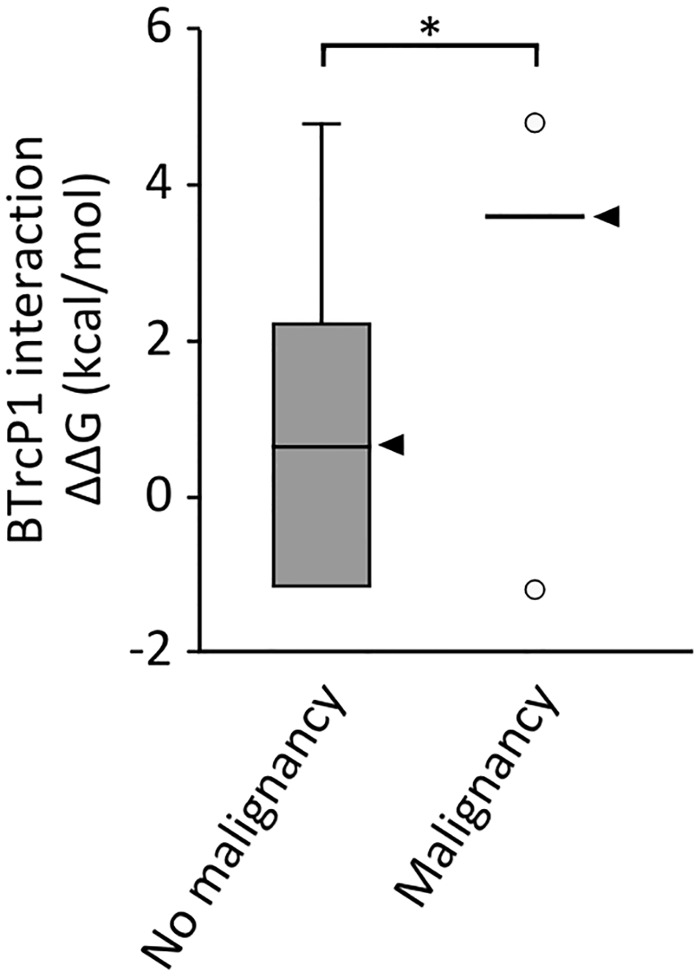
Functional effects of germline *SETBP1* mutations and risk of malignancy. Degron-βTrCP1 interaction ΔΔG for *SETBP1* mutations in individuals with SGS who did not develop a malignancy versus those who did (*p<0.05, Mann-Whitney’s U test). The median for each group is marked by an arrowhead. The criteria to be considered negative for the development of a malignancy was either reaching the age of 60 months or dying without developing a malignant tumor or leukemia.

To compare the intensity of effect of mutations in the group of individuals who developed cancer versus those who did not, we calculated the median SETBP1-βTrCP1 interaction ΔΔG value for each group based on results from *in silico* protein modeling (Figs 3B and 5). The group of individuals who developed cancer carry *SETBP1* mutations which are more disruptive to the interaction of SETBP1 with βTrCP1 than the group of patients who did not develop cancer (Fig 5; βTrCP1 interaction ΔΔG = 3.57 vs 0.65, p = 0.029, Mann-Whitney U test). Finally, we analyzed the prevalence of malignancy per genotype in 33 mutation-positive individuals with SGS for whom we had data. While the incidence of tumorigenesis is 21.2% in this pooled group, individuals with mutation D868 have a statistically significantly higher risk of developing tumors than individuals with other germline mutations in *SETBP1* (OR = 9.16, 95% CI = 1.4–59.6, p = 0.02). Remarkably, mutations in residue D868 represent the most prevalent mutation observed somatically in cancer.

## Discussion

The aims of our study were: to present the phenotype of the largest cohort of individuals with SGS and germline *SETBP1* mutations, establish genotype-phenotype correlations for germline *SETBP1* mutations and, by using these and previous findings from cancer research, provide insight into the molecular mechanism of SGS.

We present the clinical features of 47 individuals with “classic” SGS caused by germline mutations in *SETBP1* affecting the canonical degron (D868-I871). All mutations were found *de novo* in the affected proband, although two individuals in our cohort were siblings carrying the same disease-causing mutation in residue I871. This recurrence suggests parental mosaicism as the origin of the mutation but we were unable to detect the mutation in DNA from parental blood samples by deep sequencing. Interestingly, the first published report of SGS in 1978 described two siblings with the phenotype, which initially led to believe that this disorder was inherited autosomal recessively [1].

SGS is a rare but clinically recognizable developmental disorder consisting of typical facial features, neurological alterations, various congenital anomalies and increased risk of malignancy. Neurological problems often encountered include severe intellectual disability, intractable epilepsy and cerebral blindness and deafness. Individuals with SGS frequently present with congenital anomalies in multiple organ systems including heart defects, kidney and/or genital malformations and bone abnormalities. Most affected individuals do not survive past childhood due to the severity of this disorder.

Four additional individuals presented a developmental phenotype with clinical characteristics overlapping classic SGS caused by atypical *SETBP1* mutations in close proximity to the canonical degron. SGS is often recognized and diagnosed based on the reminiscent clinical features and, interestingly, the amount of clinical overlap with SGS seems to be related to the position of the mutation in relation to the canonical degron. Both individuals carrying a mutation affecting SETBP1 residue S867 had the characteristic facial features of SGS, genital anomalies and seizures but no hydronephrosis. The absence of hydronephrosis in these cases is remarkable, since it is considered one of the hallmark features of SGS [51]. However, hydronephrosis was also absent in two individuals with mutations within the degron, suggesting that it should not be recognized as an obligatory feature for the diagnosis of SGS. Although both individuals with an atypical *SETBP1* mutation in residues E862 and T873 share some features with SGS, they would not have been classified as typical SGS. In both these individuals, the mutations were detected by whole exome sequencing and lead to the diagnosis of atypical SGS *a posteriori*. Therefore, as Carvalho *et al*. recently suggested [21], previously proposed clinical diagnostic criteria for SGS may not be applicable for these cases. Due to the limited number of atypical cases, we cannot draw any conclusions on the survival and progress of the disease in these cases.

Somatic *SETBP1* mutations observed in myeloid malignancies have been shown to have a gain-of-function effect on the SETBP1 protein, leading to decreased binding of the βTrCP1 and increased protein levels [5]. This gain-of-function mechanism seems to also pertain to germline *SETBP1* mutations, as we observe that cells of individuals with germline *SETBP1* mutations have increased levels of SETBP1 protein. Furthermore, recent reports of germline chromosomal deletions and truncating mutations in *SETBP1* show that loss-of-function mutations in this gene cause a completely different phenotype from SGS [52]. Clinically, individuals with these genetic lesions present a phenotype characterized by a complete lack of expressive speech with intact receptive language abilities, decreased fine motor skills, subtle dysmorphisms and hyperactivity and autistic traits [53].

The distribution of germline *SETBP1* mutations within the canonical degron differs from that of somatic events; mutations affecting residue I871 are significantly more frequent in the germline than somatically. Furthermore, germline *SETBP1* mutations are on average less disruptive to the βTrCP1-SETBP1 interaction than somatic mutations. Notably, the isoleucine at position 871 is a variable residue within the SETBP1 degron, which is defined as “DpSGφXpT” where φ (in this case, isoleucine) represents a hydrophobic amino acid and X stands for any residue [18]. Previous studies of the structure of the conserved destruction motif in β-catenin show that the isoleucine residue in the degron has a small role in the interaction with βTrCP1, while the first three residues of the degron and the asparagine in particular are essential for protein-protein binding [18]. Thus, the molecular differences observed between germline and somatic *SETBP1* mutations are likely caused by variation in the prevalence of mutations in residue I871 in each condition. As a consequence of the weak role of residue I871 in the interaction of SETBP1 with βTrCP1, mutations in this residue, although disruptive when present in the germline, are functionally milder.

The difference in molecular consequences between somatic and germline *SETBP1* mutations is in line with findings from previous studies examining somatic and germline mutations in *PTPN11*, involved in juvenile myelomonocytic leukemia and in Noonan syndrome, respectively [54,55]. In contrast with *SETBP1* mutations, germline *PTPN11* mutations causative for Noonan syndrome rarely overlap with somatic mutations observed in leukemia. This mutual exclusivity between germline and somatic *PTPN11* mutations has been proposed to result from the existence of distinct thresholds for gain-of-function mutations in developmental phenotypes and tumorigenesis [55]. Despite their overlap, our analysis of germline and somatic *SETBP1* mutations supports this model. Malignant cell behavior in cancer can only be driven by somatic mutations with intense activation, while germline mutations with mild activation are sufficient to disrupt normal development. Consequently, gain-of-function mutations usually found somatically may lead to prenatal lethality or severe developmental alterations when present in the germline, as a result of intense activation [56]. Likewise, mild hypermorphic mutations associated with developmental disorders may be less likely to drive malignancy and are encountered less frequently as somatic events in cancer. In line with this, functional analysis of D868N, the *SETBP1* mutation most commonly found in cancer, shows that it leads to the highest increase in SETBP1 protein levels and cell proliferation, suggesting it has the strongest effect at the biochemical and cellular level. Remarkably, individuals with SGS caused by mutations in residue D868 have higher incidence of tumorigenesis with odds ratio above 9 when compared to individuals with SGS caused by other mutations. Our findings suggest that individuals with strongly activating germline mutations in *SETBP1* are at increased risk of malignancy. Due to the extremely low prevalence of SGS and the fact that malignancy is still a relatively infrequent complication of SGS, our study is limited by the small number of individuals with SGS who developed a malignancy. The correlation between strongly activating germline *SETBP1* mutations and risk of malignancy should be reproduced in a larger cohort, in order to provide accurate prognosis and personalized follow up for individuals with germline *SETBP1* mutations.

Despite the increased risk for tumorigenesis, most individuals with SGS do not develop cancer. This observation that can be extended to individuals with developmental disorders resulting from germline mutations in genes involved in cancer when mutated somatically such as *ASXL1*, *EZH2* or *ARID1A* [57–59]. As mentioned previously, this observation may be in part due to the effect of germline mutations not reaching the threshold of functional activation required to drive cancer. Furthermore, early lethality could also explain why most individuals with developmental disorders caused by germline mutations in genes involved in cancer do not develop malignancies. For instance, somatic *SETBP1* mutations have been associated mainly with chronic myeloid leukemia, a disease occurring generally in individuals above the age of 60 [5,31]. Individuals with SGS have a short life span, which may not allow for the accumulation of additional somatic mutations required for tumorigenesis. Additionally, the cellular context is also important for the expression of a mutation: certain cancer-driving mutations can only do so in the context of “aged” hematopoietic stem cells [60–62]. This combination of factors may explain why myeloid malignancy is a rare malignancy in SGS, while embryonic cancers are observed more frequently. Finally, the presence of mutations in all cells of the organism instead of a subset may eliminate cellular advantage in a single cell which would allow for clonal expansion and, eventually, malignancy.

Cell lines derived from individuals with SGS recapitulate some of the features identified in myeloid cells with *SETBP1* mutations, including increased SETBP1 and SET protein levels and enhanced cell proliferation [5,7]. Although we observed a *SETBP1* mutation-specific effect on cell proliferation, we did not detect visible differences in SET protein levels between individuals with different *SETBP1* mutations. It is possible that different *SETBP1* mutations have a subtle but distinct effect on SET protein levels that are not detectable by Western blot. However, we cannot rule out the absence of a mutation-specific effect of *SETBP1* mutations on SET protein levels with additional mutation-specific downstream alterations resulting from pathways that do not involve SET protein. In light of our findings, cell lines derived from patients with developmental disorders caused by germline mutations in genes implicated in tumorigenesis may be a valuable model for the study of downstream consequences of cancer mutations at the molecular level. Furthermore, the similarities observed in the molecular consequences of germline and somatic *SETBP1* mutations support the rationale behind recent studies which have elegantly repurposed drugs used in cancer therapy for the treatment of developmental disorders in mouse models [63]. An increase in SETBP1 protein as a result of mutations or overexpression leads to an increase in SET protein which results in the inactivation of PP2A. Although currently there are no known SETBP1 antagonists, several compounds have been described to antagonize SET protein or to lead to PP2A activation [64]. For example, OP449 is a synthetic peptide that binds to SET protein, activates PP2A and selectively inhibits cell growth in leukemia cell lines and primary patient cells [65]. Other compounds that directly activate PP2A, such as FTY720, have also been described [66]. While promising, it is yet unclear whether compounds targeting SET or PP2A may prove useful in the treatment of individuals with germline SETBP1 mutations. For one, the window of time between diagnosis and potential therapeutic interventions for early developmental phenotypes may be minimal. However, although this is still a speculative point, future therapeutic interventions may be useful to prevent progression of certain features of SGS, such as neurodegeneration [28]. Finally, it is still possible that individuals with SGS present alterations in other proteins downstream of SETBP1.

In summary, we describe the largest cohort of *SETBP1* mutation-positive SGS patients to date. Our results support that typical SGS is caused by gain-of-function mutations of *SETBP1* affecting a degron involved in SETBP1 protein stability, while novel mutations outside the canonical degron cause an atypical form of SGS characterized by a milder phenotype. We observe variability in the magnitude of effect of germline mutations within the canonical degron of SETBP1 with consequences at the biochemical level and influencing the cellular and clinical phenotype. Furthermore, our results highlight that, despite the identification of overlapping *SETBP1* mutations in SGS and myeloid malignancies, the mutation spectrum is significantly different in both conditions with functionally weaker mutations appearing predominantly as germline mutations in SGS. The parallelisms between the functional consequences of germline and somatic *SETBP1* mutations is relevant for the better understanding of SGS but could also deliver insight into the role of *SETBP1* as a cancer driver. Finally, our findings highlight that the convergence of the fields of cancer and developmental disorders uncovers common molecular mechanisms of disease for overlapping germline and somatic pathogenic mutations and may support the development of drugs with a dual therapeutic role in developmental disorders and cancer.

## Materials and methods

### Ethics statements

This study was approved by the institutional review board "Commissie Mensgebonden Onderzoek Regio Arnhem-Nijmegen" NL36191.091.11. Parents of all patients provided their written consent.

### DNA studies

Genomic DNA was extracted from saliva or blood using the QIAamp DNA Mini kit (QIAGEN). The hotspot region of *SETBP1* was amplified by PCR and Sanger sequenced. Primer sequences are listed in S6 Table.

### Phenotyping of individuals with SGS

Clinical features of participants were initially evaluated by clinicians from various countries. Photographs and medical information of all individuals were further assessed by a single clinical geneticist (BWvB). Separate informed consent was obtained for publication of photographs.

### DNA constructs

The full-length SETBP1 construct fused to a C-terminal Myc-FLAG tag was purchased from Origene (RC229443). SETBP1 variant constructs were generated using the Quikchange II Site-Directed Mutagenesis kit (Agilent). Primer sequences are listed in S5 Table. SETBP1 cDNAs were subcloned using EcoRI/XhoI restriction sites into a modified pEGFP-C2 vector (Clontech) where the N-terminal EGFP tag was replaced with a YFP tag. All constructs were verified by Sanger sequencing.

### Cell culture and transfection

Fibroblast cell lines were established from skin biopsies of SGS cases and controls. HEK293 cells and fibroblasts were cultured in DMEM supplemented with 10% fetal bovine serum (all from Invitrogen). LCLs were established by Epstein-Barr virus transformation of peripheral lymphocytes from blood samples of SGS patients and controls. LCLs were cultured in RPMI medium (Lonza) with 10% fetal bovine serum and 5% HEPES. Transfections were performed using the GeneJuice transfection reagent following the manufacturer’s instructions (Merck Millipore).

### Western blotting

Whole-cell lysates were prepared as described previously [67]. Total protein was quantified using the Pierce BCA protein assay kit (ThermoFisher Scientific). Proteins were resolved on 4–15% Tris-Glycine gels and transferred to PDVF membranes (Bio-Rad). After blotting, membranes were incubated overnight at 4°C with the appropriate primary antibodies: rabbit anti-SETBP1 (Santa Cruz, sc-85148, 1:100), rabbit anti-SET (Abcam, ab1183, 1:4000), mouse anti-β-actin (Sigma, AC-15, 1:10000) and mouse anti-FLAG (Sigma, F1804; 1:1000). Membranes were then incubated with HRP-conjugated donkey anti-rabbit (Abcam) or goat anti-mouse (Bio-Rad) secondary antibodies. Proteins were visualized using the Novex ECL Chemiluminescent Substrate Reagent kit (Invitrogen) and the ChemiDoc XRS+ System (Bio-Rad).

### Protein stability assays

Cells were transfected in clear-bottomed black 96-well plates in hexaplicate with YFP-SETBP1 expression plasmids together with a modified pmCherry-C1 plasmid to normalize for transfection efficiency. Fourteen hours post-transfection, YFP and mCherry fluorescence intensities were measured for 24h in live cells in a TECAN M200PRO microplate reader at 37°C and 5% CO_2_. In the case of stability assays with proteasome inhibitor, MG132 (Sigma) was added to the culture medium (10uM final concentration) 48 hours post-transfection and fluorescence intensities were measured at 0, 3 and 12 hours.

### Fluorescence microscopy

Cells were grown on poly-L-lysine (Sigma) coated coverslips and were fixed 48 hours post-transfection with 4% paraformaldehyde (Electron Microscopy Sciences) for 10 minutes at room temperature. YFP was visualized by direct fluorescence. Nuclei were visualized with DAPI (Vectorlabs). Fluorescence images were obtained using an Axio Imager Z1 fluorescence microscope (Zeiss).

### Proliferation assays

Lymphoblastoid cell lines were synchronized by overnight serum starvation, after which they were seeded in 24-well plates at a concentration of 160,000 cells/mL (three replicates per cell line). A measurement was performed every 24 hours, in which the cells were mixed with Trypan blue and counted.

### Modeling of protein in Yasara and FoldX

The SETBP1 degron variants observed in SGS and in leukemia were manually curated from previously published reports) [4–7,23–27,30–48]. Mutations were modeled using the YASARA structural simulation software (http://www.yasara.org/). The protein model for βTrCP1 and degron sequence from β-catenin was obtained from the RCSB protein bank (1p22) [18]. The FoldX plugin for YASARA was used to calculate ΔΔG values [68].

### RNA-sequencing

LCLs from individuals with SGS were derived from blood samples of individuals with germline *SETBP1* mutations D868N or I871T (n = 2 for each). LCLs from controls (n = 8) were derived from blood samples of individuals with different forms of intellectual disability [69]. RNA was purified from cell cultures using RNeasy mini kit from QIAGEN. RNAseq libraries were prepared using the TruSeq Stranded mRNA kit (Illumina) and sequenced on a Nextseq platform using 2x75bp paired end sequencing. RNA-seq samples were mapped using Kallisto (version 0.42.4) to the human genome (hg19.75). At least 70% of the reads for each sample were mapped to the human genome entailing at least 39.6 million reads in the sample with the lowest sequencing depth. Differential gene expression was performed using DESeq2 [49]. Gene set enrichment analysis was performed using the GSEA software [50].

### Statistics

Statistical analysis was performed using R Statistical Software (http://www.r-project.org/ version 3.1.2, R Foundation for Statistical Computing, Vienna, Austria). Student’s T test was used for comparison of two groups with normal distribution, otherwise Mann-Whitney’s U test was used. For comparison of multiple groups against each other, we used ANOVA and Tukey’s test. To determine whether the distribution of mutations within the degron was the same in germline and somatic mutations, we used Fisher’s test and performed Bonferroni multiple test correction.

## Supporting information

S1 Table Table with all clinical information from 51 individuals with gain-of-function *de novo* mutations within or in close proximity to the *SETBP1* degron. Of these, 26 are novel cases of classic SGS caused by mutations affecting SETBP1 residues D868-I871 and 3 are novel individuals presenting an atypical form of SGS secondary to mutations affecting SETBP1 residues E862, S867 and T873.
(XLSX)

S2 Table Protein sequence alignment of degrons targeted by βTrCP. Based on Low TY et al. A systems-wide screen identifies substrates of the SCF bTRCP ubiquitin ligase. Sci Signal 2014; 7: 1–12
(PDF)

S3 Table ΔΔG values for protein stability and degron-βTrCP1 interaction for SETBP1 variants reported in SGS cases and in myeloproliferative disorders. (PDF)

S4 Table Table with germline and somatic *SETBP1* mutations reported in literature. Germline *SETBP1* mutations were retrieved from previous reports from mutation-positive individuals with SGS. Two siblings with the same germline *SETBP1* mutation were identified in our cohort, suggesting parental mosaicism for the pathogenic mutation. These two mutations were counted as a single event. Somatic *SETBP1* mutations were retrieved from the Catalogue of Somatic Mutations in Cancer (COSMIC) database and the cited references were manually curated to avoid double counts for a single mutation (*e*.*g*. sample from same patient at diagnosis versus relapse).
(XLSX)

S5 Table Gene set enrichment analysis for genes differentially expressed in LCLs from individuals with germline *SETBP1* mutations D868N or I871T and LCLs from controls. Gene set enrichment analysis was performed as described in Subramanian et al. *PNAS* 2005 using curated gene sets from the reactome pathway database. An enrichment is observed for genes involved in mRNA transcription and translation, mitochondrial respiration and in the cell cycle. While no enrichment was observed for specific pathways, a closer examination of the genes involved in cell cycle that we detected to be enriched in the samples of individuals with germline *SETBP1* mutations revealed that the genes identified were associated with mitosis, sister chromatid cohesion, DNA replication and nucleosome assembly.
(PDF)

S6 Table Sequences of primers used in this study. (PDF)

S1 Fig Facial features in typical Schinzel-Giedion syndrome. Panels A to I show the facial features of patients with germline SETBP1 mutations within the canonical degron leading to typical SGS (A: current case 1; B: current case 2; C: current case 3; D: current case 12; E: current case 16; F: current case 18; G current case 20; H current case 23). Progression of facial features with age in current case 9 with germline SETBP1 mutation G870S at 1 week of age (I), at 1 ½ years of age (J), at 6 years of age (K) and at 7 years of age (L).
(TIF)

S2 Fig LogRank survival analysis performed for 44 individuals with SGS for which data was available from our own cohort and previous reports. Dots represent censored data, for age at which the individual was last known to be alive. The group of patients with mutations in residue G870 had a statistically significantly longer survival than individuals with mutations in residue D868 (median of 60 months versus 24 months respectively, p<0.01).
(TIF)

S3 Fig Phenotype of individuals with germline *SETBP1* mutations outside the canonical degron leading to atypical Schinzel-Giedion. Individual with germline SETBP1 mutation S867R (current case 27) at 1 week of age (A), at nine months (B), at two years of age (C) and at four years of age (D). Patient with germline SETBP1 mutation E862K (current case 28) at 5 years of age (E and F). Right hand and foot (G and H), note the small feet with short toes. Individual with germline SETBP1 mutation T873I (current case 29) at one month of age (I), at eleven months (J), at three years of age (K) and at four years of age (L).
(TIF)

S4 Fig Fluorescence imaging of HEK293 cells expressing YFP-tagged SETBP1 variants (green). Nuclei were stained with DAPI (blue). Wild-type (WT) SETBP1 and disease-causing SETBP1 variants were expressed in HEK293 cells as YFP fusion proteins. Direct fluorescence imaging of SETBP1 variants showed that the WT protein localizes to the nucleus, with a speckle-like pattern typical of chromatin-interacting proteins. Pathogenic SETBP1 protein variants occurring within (D868N, I871) or in close proximity (E862K) to the canonical degron sequence had no effect on protein localization.
(TIF)

S5 Fig Relative expression of SETBP1 protein variants in live HEK293 cells treated with 50ug/uL cycloheximide or vehicle only as controls. Bars represent the standard error. (* p<0.05, ** p<0.01, *** p<0.001 versus cells at same time point treated with vehicle; Student’s T test and Mann-Whitney U test for measurements with non-normal distribution). The fold decrease in relative expression at 24 hours is significantly higher for wild-type SETBP1 compared to all variants.
(TIF)

S6 Fig Densitometry for Western blot of overexpressed wild type and mutant SETBP1-FLAG in HEK293 cells using actin for normalization. (TIF)

S7 Fig Levels of *SETBP1* mRNA in fibroblasts and Lymphoblastoid Cell Lines (LCLs) from individuals with SGS as compared to controls. *SETBP1* mRNA levels were normalized to *ACTB* and *GAPDH*. The results shown represent the mRNA levels in fibroblasts cells lines from 2 controls, fibroblast cell lines from 2 individuals with SGS, LCLs from 2 controls and LCLs from 3 individuals with SGS. The bars represent the standard error. NS: not significant. ** p<0.01, Student’s T test. The decrease in *SETBP1* mRNA levels observed in individuals with SGS compared to controls could be the result of a feedback mechanism in which increased SETBP1 protein levels may lead to a reduction in *SETBP1* transcription thereby decreasing SETBP1 protein levels. A similar observation was reported for myeloid progenitor cells immortalized by mutant *SETBP1* compared to cells immortalized by wild-type *SETBP1* in Makishima H. *et al*. Nat Genetics 2013. We speculate that this mechanism would be able to lower SETBP1 protein to normal levels for SETBP1 harboring weaker mutations, such as I871T, but not for stronger mutations, such as D868N.
(TIF)

S8 Fig Heatmap of RNAseq transcriptome analysis showing differential gene expression between LCLs from controls (n = 8) and LCLs from individuals with germline *SETBP1* mutations D868N or I871T (n = 2 for each). Only the top 250 most significant genes are shown in this figure. Yellow indicates high expression while red indicates low expression. Differential gene expression was performed using DESEq2, which showed differential expression of 1811 genes (adjusted p-value <0.01), of which 632 are upregulated and 1179 are downregulated.
(TIF)
