# Supplementary material for: Overlapping SETBP1 gain-of-function mutations in Schinzel-Giedion syndrome and hematologic malignancies
Source: PLoS Genet. 2017 Mar 27;13(3):e1006683. doi: 10.1371/journal.pgen.1006683 (PMC5386295; doi:10.1371/journal.pgen.1006683)
Supplement: S2 Table — Based on Low TY et al. A systems-wide screen identifies substrates of the SCF bTRCP ubiquitin ligase. Sci Signal 2014; 7: 1–12 (PDF) [file pgen.1006683.s002.pdf]

Gene name	Uniprot accession number	Sequence of βTrCP binding site (DpSGϕXpS/pT)	Motif start position	Motif stop position
SETBP	Q9Y6X0	DSGIGT	868	873
CTNNB1	P35222	DSGIHS	32	37
NFE2L2	Q16236	DSGISL	343	348
ATF4	P18848	DSGICM	218	223
EEF2K	O00418	DSGYPS	440	445
CLSPN	Q9HAW4	DSGQGS	29	34
REST	Q13127	DEGIHS	1008	1013
CDC25A	P30304	DSGFCL	81	86
PDCD4	Q53EL6	DSGRGD	70	75
PER1	O15534	TSGCSS	121	126
