# Supplementary material for: Overlapping SETBP1 gain-of-function mutations in Schinzel-Giedion syndrome and hematologic malignancies
Source: PLoS Genet. 2017 Mar 27;13(3):e1006683. doi: 10.1371/journal.pgen.1006683 (PMC5386295; doi:10.1371/journal.pgen.1006683)
Supplement: S3 Table — (PDF) [file pgen.1006683.s003.pdf]

Mutation	Stability ΔΔG (kcal/mol)	Standard deviation	βTrCP1 Interaction ΔΔG (kcal/mol)	Standard deviation	Interpretation	Germline	Somatic
D868A	1.08	0.24	2.78	0.14	Destabilizing	Y	N
D868G	0.99	0.21	2.72	0.21	Destabilizing	N	Y
D868H	9.98	3.04	10.77	3.43	Highly destabilizing	N	Y
D868N	2.30	0.17	3.57	0.49	Highly destabilizing	Y	Y
D868Y	12.31	2.88	11.74	2.77	Highly destabilizing	Y	Y
S869G	0.10	0.11	0.06	0.11	Neutral	N	Y
S869N	-0.25	0.12	-0.47	0.21	Neutral	Y	Y
S869R	-0.97	0.16	-1.05	0.17	Neutral	Y	Y
G870C	1.27	1.04	0.79	0.98	Destabilizing	Y	N
G870D	4.94	1.46	4.78	1.48	Highly destabilizing	Y	Y
G870R	11.72	1.81	10.84	2.02	Highly destabilizing	N	Y
G870S	1.07	0.51	0.65	0.50	Destabilizing	Y	Y
G870V	5.40	2.12	5.21	2.05	Highly destabilizing	N	Y
I871S	0.17	0.31	-0.38	0.34	Neutral	Y	Y
I871T	-0.22	0.42	-1.15	0.34	Neutral	Y	Y
