# Supplementary material for: Overlapping SETBP1 gain-of-function mutations in Schinzel-Giedion syndrome and hematologic malignancies
Source: PLoS Genet. 2017 Mar 27;13(3):e1006683. doi: 10.1371/journal.pgen.1006683 (PMC5386295; doi:10.1371/journal.pgen.1006683)
Supplement: S5 Table — Gene set enrichment analysis was performed as described in Subramanian et al. PNAS 2005 using curated gene sets from the reactome pathway database. An enrichment is observed for genes involved in mRNA transcription and translation, mitochondrial respiration and in the cell cycle. While no enrichment was observed for specific pathways, a closer examination of the genes involved in cell cycle that we detected to be enriched in the samples of individuals with germline SETBP1 mutations revealed that the genes identified were associated with mitosis, sister chromatid cohesion, DNA replication and nucleosome assembly. (PDF) [file pgen.1006683.s005.pdf]

NAME	SIZE	ES	NES	FDR q-val	FWER p-val
REACTOME PEPTIDE CHAIN ELONGATION	100	0.54	6.55	<0.00001	<0.001
REACTOME INFLUENZA VIRAL RNA TRANSCRIPTION AND REPLICATION	116	0.47	6.08	<0.00001	<0.001
REACTOME 3 UTR MEDIATED TRANSLATIONAL REGULATION	121	0.46	5.82	<0.00001	<0.001
REACTOME NONSENSE MEDIATED DECAY ENHANCED BY THE EXON JUNCTION COMPLEX	121	0.44	5.62	<0.00001	<0.001
REACTOME TRANSLATION	163	0.35	5.44	<0.00001	<0.001
REACTOME SRP DEPENDENT COTRANSLATIONAL PROTEIN TARGETING TO MEMBRANE	124	0.42	5.32	<0.00001	<0.001
REACTOME INFLUENZA LIFE CYCLE	150	0.35	4.95	<0.00001	<0.001
REACTOME METABOLISM OF PROTEINS	427	0.16	3.95	<0.00001	<0.001
REACTOME FORMATION OF THE TERNARY COMPLEX AND SUBSEQUENTLY THE 43S COMPLEX	53	0.44	3.80	<0.00001	<0.001
REACTOME ACTIVATION OF THE MRNA UPON BINDING OF THE CAP BINDING COMPLEX AND EIFS AND SUBSEQUENT BINDING TO 43S	61	0.40	3.75	<0.00001	<0.001
REACTOME METABOLISM OF MRNA	224	0.20	3.49	<0.00001	<0.001
REACTOME RESPIRATORY ELECTRON TRANSPORT ATP SYNTHESIS BY CHEMIOSMOTIC COUPLING AND HEAT PRODUCTION BY UNCOUPLING PROTEINS	80	0.33	3.42	<0.00001	<0.001
REACTOME TCA CYCLE AND RESPIRATORY ELECTRON TRANSPORT	116	0.27	3.40	<0.00001	<0.001
REACTOME CHOLESTEROL BIOSYNTHESIS	21	0.62	3.39	<0.00001	<0.001
REACTOME METABOLISM OF RNA	269	0.17	3.30	<0.00001	<0.001
REACTOME RESPIRATORY ELECTRON TRANSPORT	65	0.33	3.18	<0.00001	<0.001
REACTOME TELOMERE MAINTENANCE	73	0.32	3.15	<0.00001	<0.001
REACTOME GENERIC TRANSCRIPTION PATHWAY	323	0.14	2.94	1.28E-04	0.002
REACTOME CELL CYCLE	396	0.13	2.90	1.22E-04	0.002
REACTOME GLYCOLYSIS	25	0.48	2.89	1.15E-04	0.002
REACTOME CHROMOSOME MAINTENANCE	116	0.23	2.84	2.28E-04	0.004
REACTOME RNA POL I PROMOTER OPENING	59	0.30	2.75	3.28E-04	0.006
REACTOME GLUCONEOGENESIS	29	0.41	2.63	7.19E-04	0.014
REACTOME MEIOTIC RECOMBINATION	80	0.25	2.59	0.001	0.024
REACTOME PACKAGING OF TELOMERE ENDS	46	0.32	2.53	0.001	0.039
REACTOME DEPOSITION OF NEW CENPA CONTAINING NUCLEOSOMES AT THE CENTROMERE	61	0.27	2.51	0.002	0.05
