# Supplementary material for: Overlapping SETBP1 gain-of-function mutations in Schinzel-Giedion syndrome and hematologic malignancies
Source: PLoS Genet. 2017 Mar 27;13(3):e1006683. doi: 10.1371/journal.pgen.1006683 (PMC5386295; doi:10.1371/journal.pgen.1006683)
Supplement: S6 Table — (PDF) [file pgen.1006683.s006.pdf]

Name	Sequence (5'-3')	Purpose
SETBP1 Exon4 F	CTTCACCAGCAGCTATGCAC	Sanger sequencing
SETBP1 Exon4 R	CGGTGGGAGATTCTGAACAC	Sanger sequencing
SETBP1 E862K F	GAGTCCCACAGTAAGGAGACGATCCC	Site-directed mutagenesis
SETBP1 E862K R	GGGGATCGTCTCCTTACTGTGGGACTC	Site-directed mutagenesis
SETBP1 D868N F	GACGATCCCCAGCAACAGCGGCATTGG	Site-directed mutagenesis
SETBP1 D868N R	CCAATGCCGCTGTTGCTGGGGATCGTC	Site-directed mutagenesis
SETBP1 S869N F	GACGATCCCCAGCGACAACGGCATTGG	Site-directed mutagenesis
SETBP1 S869N R	CCAATGCCGTTGTCGCTGGGGATCGTC	Site-directed mutagenesis
SETBP1 G870S F	AGCGACAGCAGCATTGGGACAGAC	Site-directed mutagenesis
SETBP1 G870S R	GTCTGTCCCAATGCTGCTGTCGCT	Site-directed mutagenesis
SETBP1 I871T F	GACAGCGGCACTGGGACAGACAAC	Site-directed mutagenesis
SETBP1 I871T R	GTTGTCTGTCCCAGTGCCGCTGTC	Site-directed mutagenesis
