# Supplementary figures and images for: Overlapping SETBP1 gain-of-function mutations in Schinzel-Giedion syndrome and hematologic malignancies
Source: PLoS Genet. 2017 Mar 27;13(3):e1006683. doi: 10.1371/journal.pgen.1006683 (PMC5386295; doi:10.1371/journal.pgen.1006683)

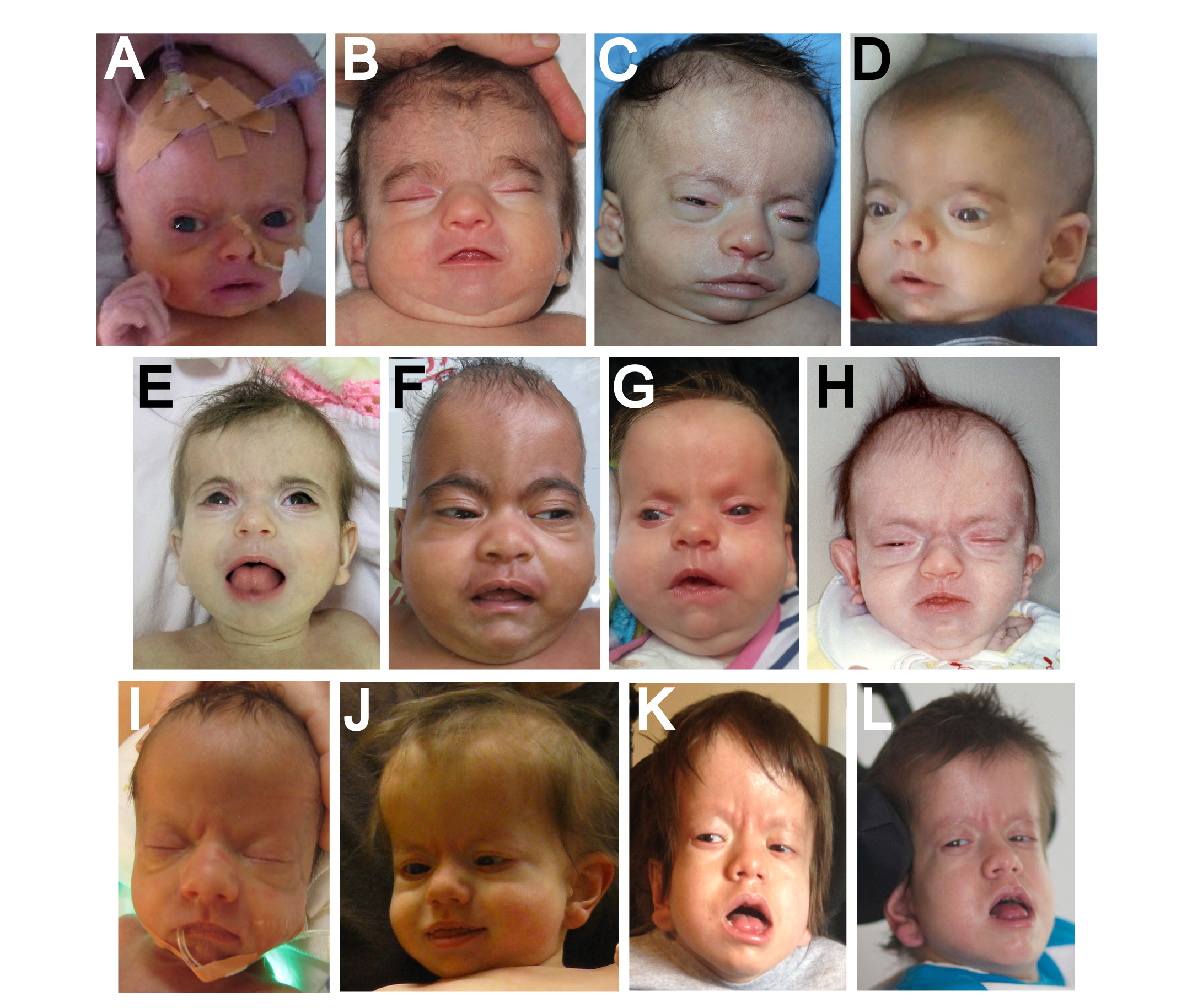

Supplement: S1 Fig — Panels A to I show the facial features of patients with germline SETBP1 mutations within the canonical degron leading to typical SGS (A: current case 1; B: current case 2; C: current case 3; D: current case 12; E: current case 16; F: current case 18; G current case 20; H current case 23). Progression of facial features with age in current case 9 with germline SETBP1 mutation G870S at 1 week of age (I), at 1 ½ years of age (J), at 6 years of age (K) and at 7 years of age (L). (TIF) [file pgen.1006683.s007.tif]

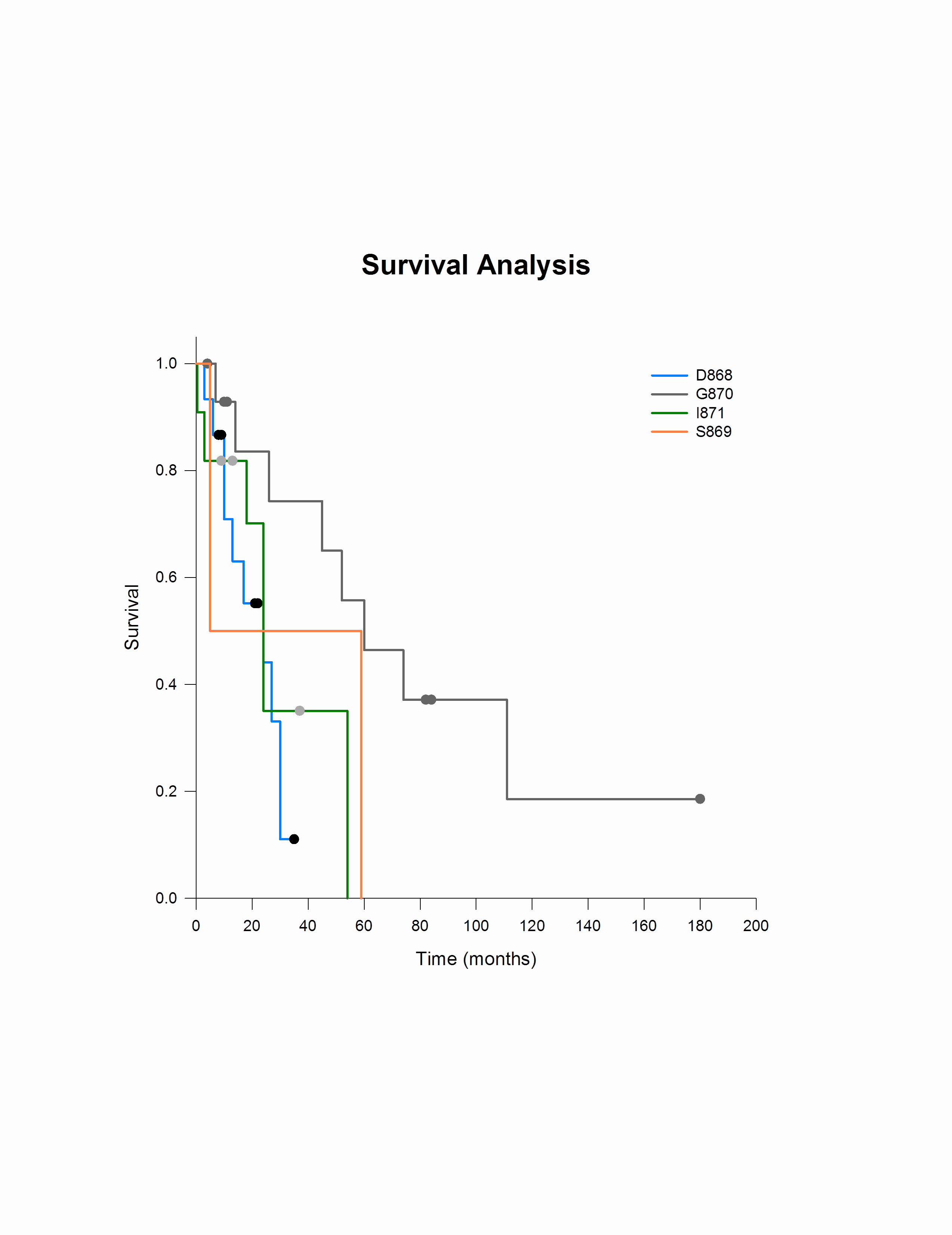

Supplement: S2 Fig — Dots represent censored data, for age at which the individual was last known to be alive. The group of patients with mutations in residue G870 had a statistically significantly longer survival than individuals with mutations in residue D868 (median of 60 months versus 24 months respectively, p<0.01). (TIF) [file pgen.1006683.s008.TIF]

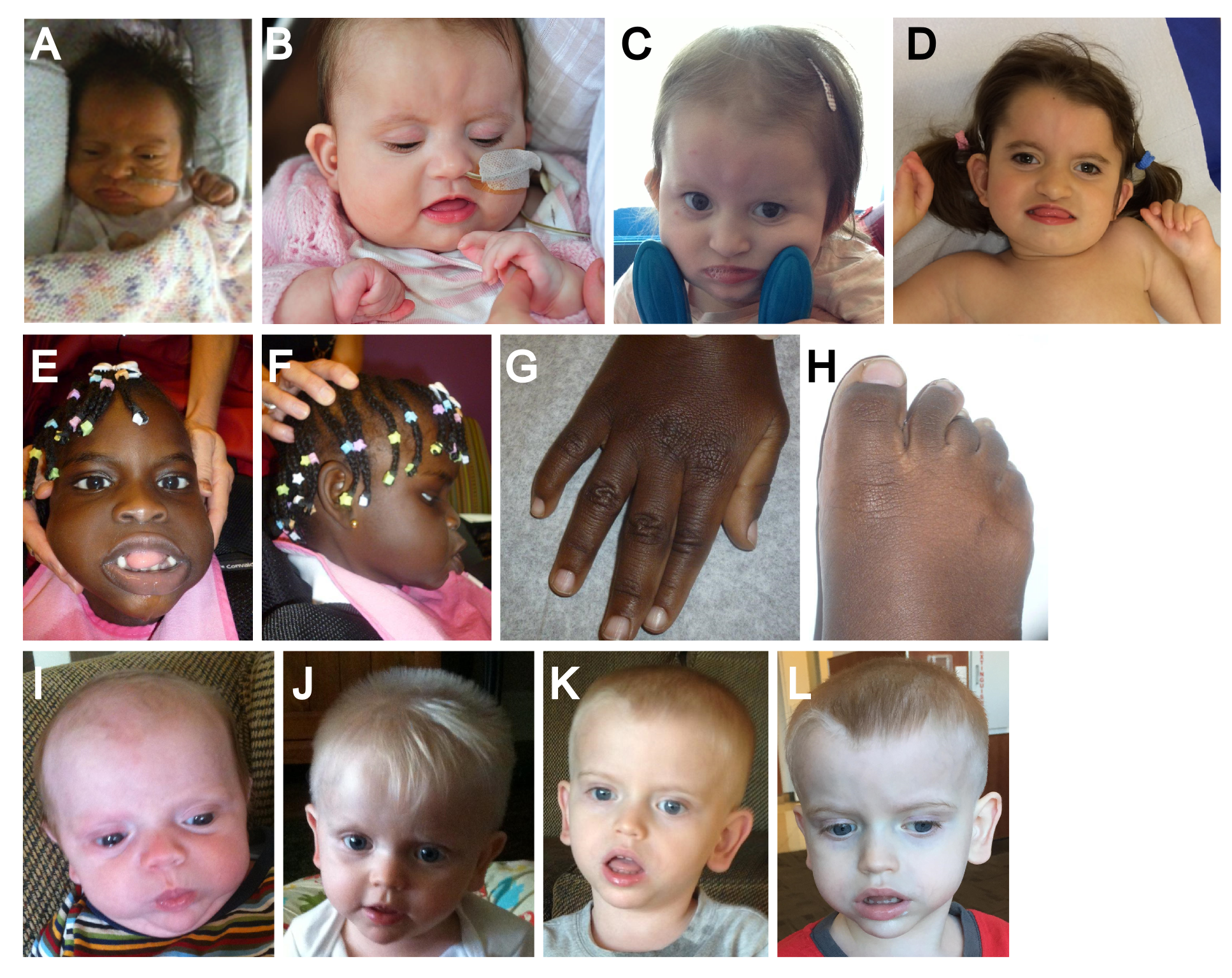

Supplement: S3 Fig — Individual with germline SETBP1 mutation S867R (current case 27) at 1 week of age (A), at nine months (B), at two years of age (C) and at four years of age (D). Patient with germline SETBP1 mutation E862K (current case 28) at 5 years of age (E and F). Right hand and foot (G and H), note the small feet with short toes. Individual with germline SETBP1 mutation T873I (current case 29) at one month of age (I), at eleven months (J), at three years of age (K) and at four years of age (L). (TIF) [file pgen.1006683.s009.tif]

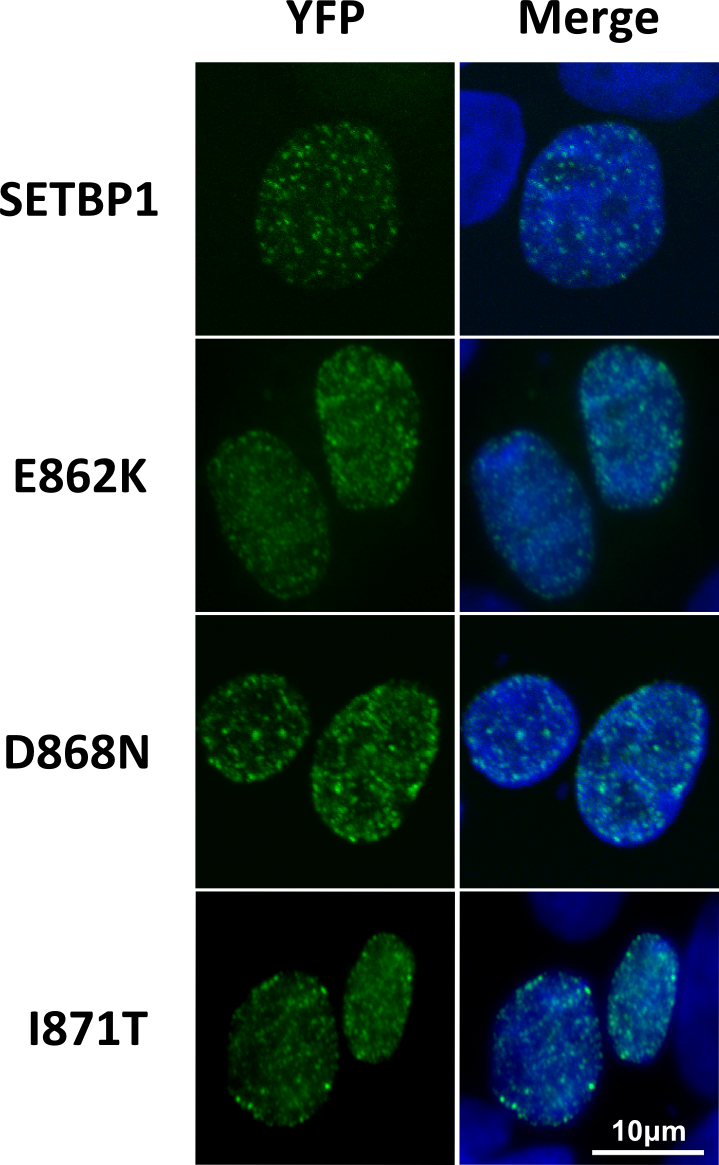

Supplement: S4 Fig — Nuclei were stained with DAPI (blue). Wild-type (WT) SETBP1 and disease-causing SETBP1 variants were expressed in HEK293 cells as YFP fusion proteins. Direct fluorescence imaging of SETBP1 variants showed that the WT protein localizes to the nucleus, with a speckle-like pattern typical of chromatin-interacting proteins. Pathogenic SETBP1 protein variants occurring within (D868N, I871) or in close proximity (E862K) to the canonical degron sequence had no effect on protein localization. (TIF) [file pgen.1006683.s010.tif]

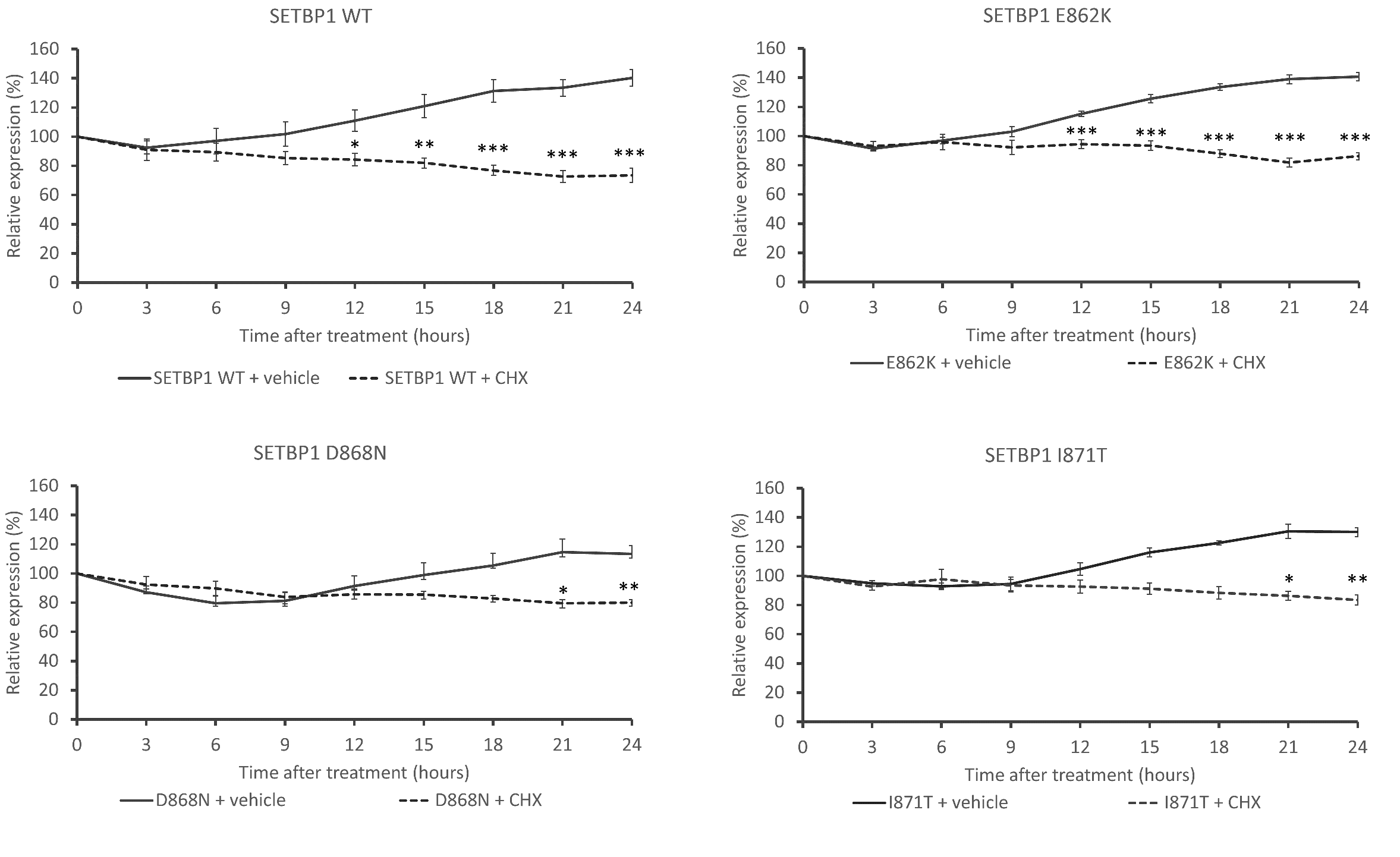

Supplement: S5 Fig — Bars represent the standard error. (* p<0.05, ** p<0.01, *** p<0.001 versus cells at same time point treated with vehicle; Student’s T test and Mann-Whitney U test for measurements with non-normal distribution). The fold decrease in relative expression at 24 hours is significantly higher for wild-type SETBP1 compared to all variants. (TIF) [file pgen.1006683.s011.tif]

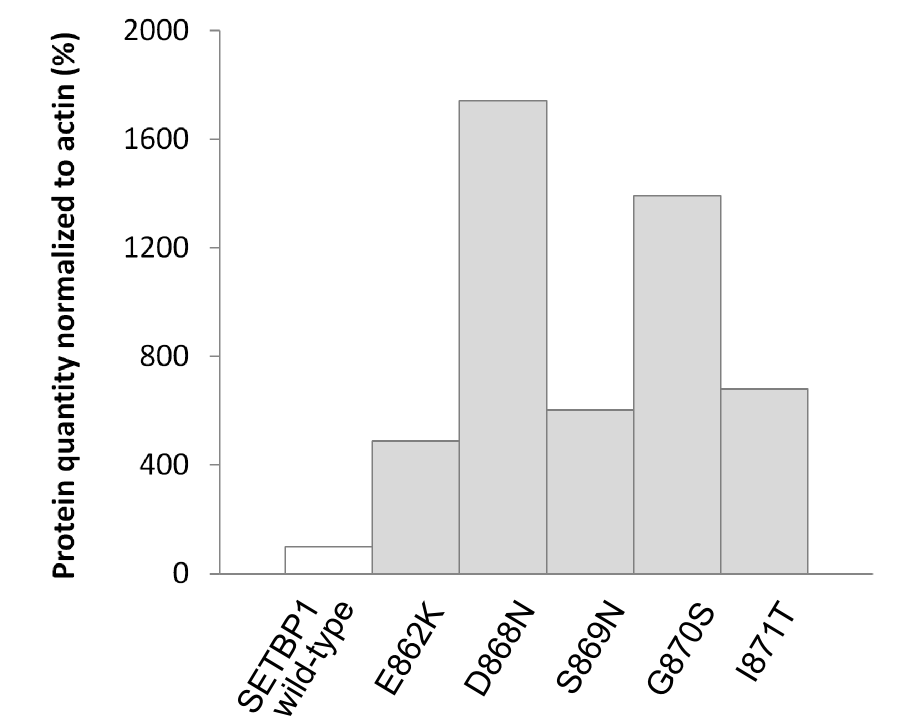

Supplement: S6 Fig — (TIF) [file pgen.1006683.s012.tif]

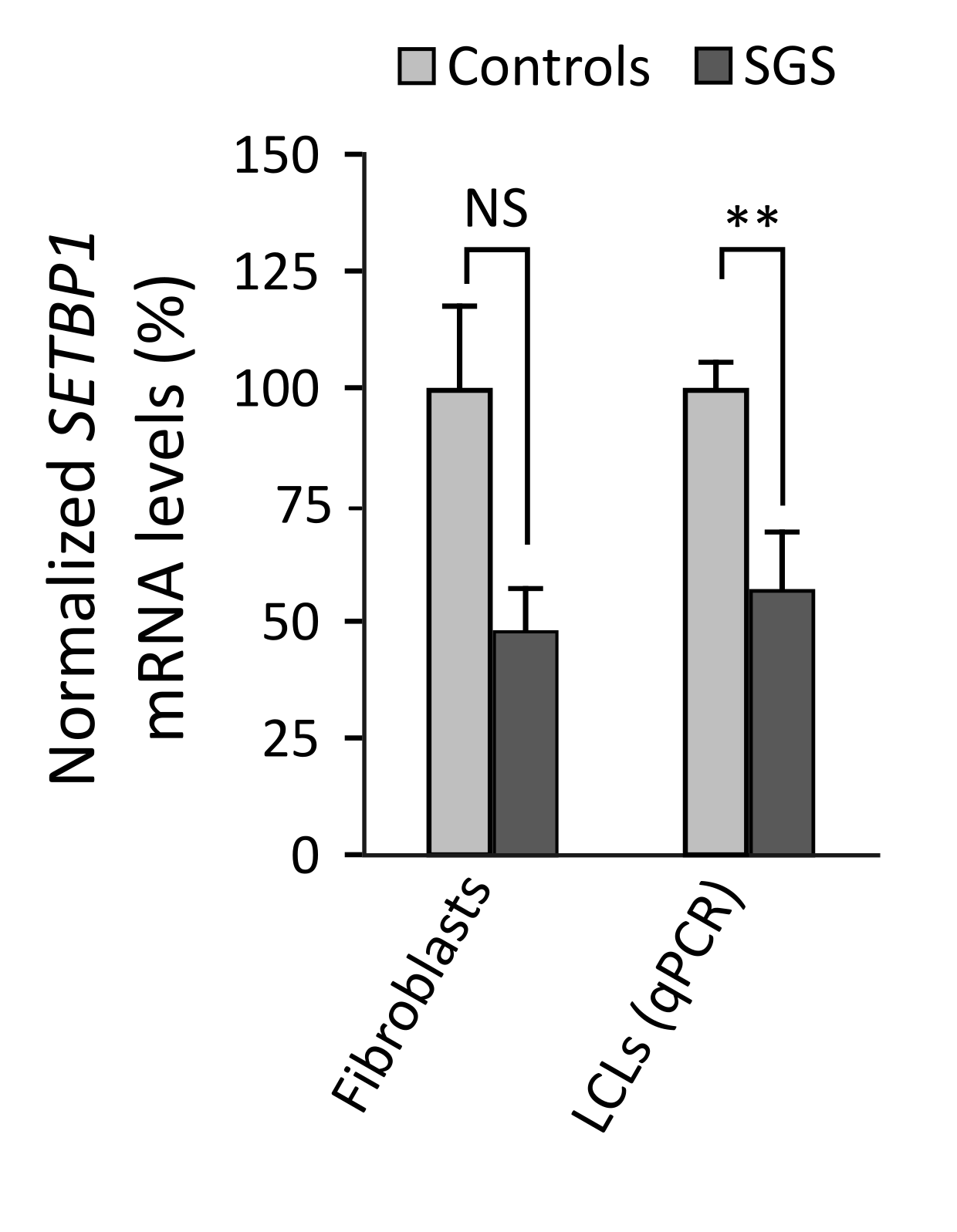

Supplement: S7 Fig — SETBP1 mRNA levels were normalized to ACTB and GAPDH. The results shown represent the mRNA levels in fibroblasts cells lines from 2 controls, fibroblast cell lines from 2 individuals with SGS, LCLs from 2 controls and LCLs from 3 individuals with SGS. The bars represent the standard error. NS: not significant. ** p<0.01, Student’s T test. The decrease in SETBP1 mRNA levels observed in individuals with SGS compared to controls could be the result of a feedback mechanism in which increased SETBP1 protein levels may lead to a reduction in SETBP1 transcription thereby decreasing SETBP1 protein levels. A similar observation was reported for myeloid progenitor cells immortalized by mutant SETBP1 compared to cells immortalized by wild-type SETBP1 in Makishima H. et al. Nat Genetics 2013. We speculate that this mechanism would be able to lower SETBP1 protein to normal levels for SETBP1 harboring weaker mutations, such as I871T, but not for stronger mutations, such as D868N. (TIF) [file pgen.1006683.s013.tif]

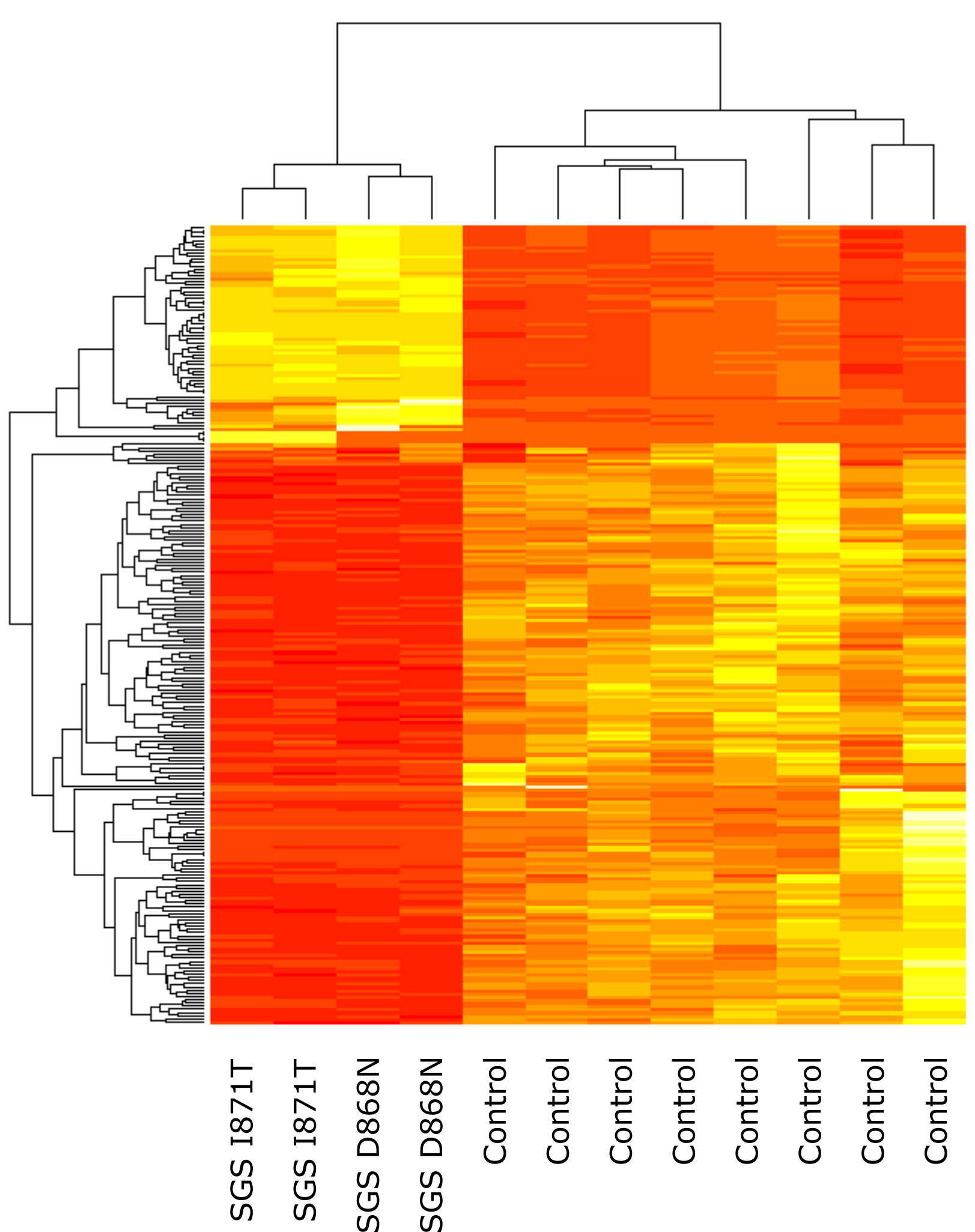

Supplement: S8 Fig — Only the top 250 most significant genes are shown in this figure. Yellow indicates high expression while red indicates low expression. Differential gene expression was performed using DESEq2, which showed differential expression of 1811 genes (adjusted p-value <0.01), of which 632 are upregulated and 1179 are downregulated. (TIF) [file pgen.1006683.s014.tif]
